# Use of Curcumin, a Natural Polyphenol for Targeting Molecular Pathways in Treating Age-Related Neurodegenerative Diseases

**DOI:** 10.3390/ijms19061637

**Published:** 2018-05-31

**Authors:** Panchanan Maiti, Gary L. Dunbar

**Affiliations:** 1 Field Neurosciences Institute Laboratory for Restorative Neurology, Central Michigan University, Mt. Pleasant, MI 48859, USA; 2 Program in Neuroscience, Central Michigan University, Mt. Pleasant, MI 48859, USA; 3 Department of Psychology, Central Michigan University, Mt. Pleasant, MI 48859, USA; 4 Field Neurosciences Institute, St. Mary’s of Michigan, Saginaw, MI 48604, USA; 5 Department of Biology, Saginaw Valley State University, Saginaw, MI 48610, USA; 6 Brain Research Laboratory, Saginaw Valley State University, Saginaw, MI 48610, USA

**Keywords:** neurodegenerative diseases, amyloidosis, curcumin, neuroinflammation, anti-amyloid, molecular chaperones, natural polyphenol

## Abstract

Progressive accumulation of misfolded amyloid proteins in intracellular and extracellular spaces is one of the principal reasons for synaptic damage and impairment of neuronal communication in several neurodegenerative diseases. Effective treatments for these diseases are still lacking but remain the focus of much active investigation. Despite testing several synthesized compounds, small molecules, and drugs over the past few decades, very few of them can inhibit aggregation of amyloid proteins and lessen their neurotoxic effects. Recently, the natural polyphenol curcumin (Cur) has been shown to be a promising anti-amyloid, anti-inflammatory and neuroprotective agent for several neurodegenerative diseases. Because of its pleotropic actions on the central nervous system, including preferential binding to amyloid proteins, Cur is being touted as a promising treatment for age-related brain diseases. Here, we focus on molecular targeting of Cur to reduce amyloid burden, rescue neuronal damage, and restore normal cognitive and sensory motor functions in different animal models of neurodegenerative diseases. We specifically highlight Cur as a potential treatment for Alzheimer’s, Parkinson’s, Huntington’s, and prion diseases. In addition, we discuss the major issues and limitations of using Cur for treating these diseases, along with ways of circumventing those shortcomings. Finally, we provide specific recommendations for optimal dosing with Cur for treating neurological diseases.

## 1. Introduction

Aggregation of misfolded amyloid proteins and their deposition in intracellular and extracellular spaces of the central nervous system (CNS) are associated with several neurological diseases, including Alzheimer’s (AD), Parkinson’s (PD), Huntington’s (HD) and prion diseases [1,2]. Most of these diseases are age-related, complicated disorders which involve a multitude of causative factors, including neuroinflammation [3], oxidative damage and deposition of misfolded protein aggregates [4]. These events can occur separately or together or in causing neuronal degeneration, which leads to perturbation of neuronal communications, resulting in long-term cognitive and motor dysfunction. The neuropathological onset of these diseases may have occurred long before the manifestation of overt symptoms, which underscores the need for early diagnosis and therapy. Although there have been several studies using therapeutic strategies involving anti-amyloids, anti-inflammatory agents, and small molecule drugs [5], to slow or halt the progression of neurological diseases, none of them have proven effective without serious effects or abbreviated half-lives. As a potent anti-amyloid natural polyphenol, curcumin (Cur) has gained considerable attention as a promising therapeutic agent for AD and other complicated neurological diseases [6,7]. Although Cur has been considered a wonder molecule for use in Indian and Southeast Asian traditional Ayurvedic medicine for a very long time, primarily due to its anti-inflammatory or wound-healing properties [8], its anti-amyloidogenic properties have only been discovered recently [9]. Cur binds to, and inhibits, amyloid-beta protein (Aβ) aggregation, and improves motor coordination and cognition in animal models of AD and other neurodegenerative diseases [9,10]. However, the poor water solubility, instability in body fluids, rapid degradation, and limited bioavailability has curtailed the use of Cur as a therapeutic for neurological diseases [11].

The outlook for using Cur as a therapeutic agent has changed dramatically with the discovery of new formulations for Cur, including liposome-Cur, Cur-conjugated with nanogel, dendrimer-Cur, Cur with silver, or gold nanoparticles and Cur in solid lipid nanoparticles (SLN) [11]. The SLN formula of Cur (nanoCur) has been shown to increase its bioavailability and therapeutic value for neurological diseases [12,13,14]. This review article addresses the basic understanding of the molecular signaling mechanisms of Cur therapy and its potential impact on major neurological diseases, such as AD, PD, HD, and prion diseases, along with recent findings from our laboratory.

## 2. Curcumin: The Major Active Polyphenol of Turmeric

### 2.1. Source

**Figure 1:**
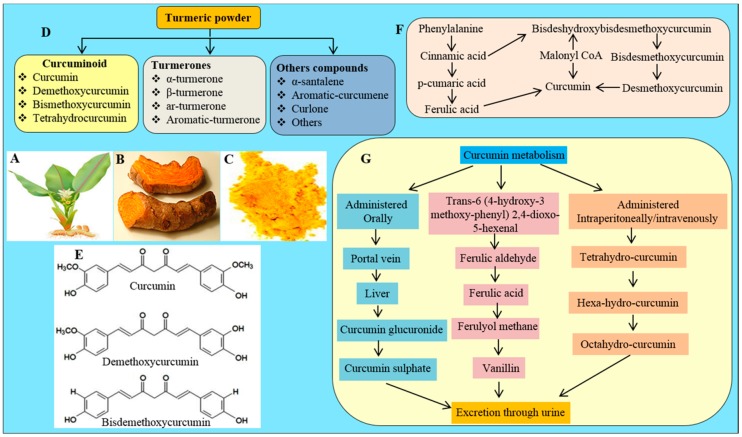
Chemical structure of Cur and its derivatives. (**A**–**C**) *Curcuma longa*, its rhizomes and turmeric extract; (**D**) Different chemical components of turmeric extract; (**E**) Chemical structure of principal ingredients of curcuminoid; (**F**) pathway of Cur-biosynthesis; and (**G**) Cur metabolism in our body.

The rhizomes of the *Curcuma longa* (family: *Zingiberaceae*) herb is the source of turmeric (Figure 1A–C). The principal yellow pigment present in the turmeric root is Cur (Figure 1E), which was identified in early 1900 by Lampe and Milobedzka. Its structure and biochemical analyses revealed that about 2.5–6% of turmeric contains pure Cur [15] (Table 1). The commercial turmeric extract contains many other components, including three main types of curcuminoids, such as (a) Cur-I (diferuloylmethane, ~77%); (b) Cur-II (demethoxyCur, DMC, ~17%); and (c) Cur-III (bisdemethoxyCur, BDMC, ~3%) (Figure 1). In addition, four identified turmerones (α-turmerone, β-turmerone, ar-turmerone, and aromatic-turmerone), as well as α-santalene, aromatic-curcumene, curlone, and other compounds were also found in turmeric extract (Figure 1D).

### 2.2. Chemistry of Cur

Cur is a natural polyphenol, chemically known as diferuloylmethane (C_21_H_20_O_6_), with molecular mass of 368.37 g/mol. Its International Union of Pure and Applied Chemistry (IUPAC) name is 1,7-bis (4-hydroxy-3-methoxy phenyl)-1,6-heptadiene-3,5-dione. There are two aryl rings containing orthomethoxy phenolic OH-groups, which are symmetrically linked to a β-diketone moiety (Figure 1E). The melting point of Cur is ~183 °C. Cur can co-exist with several tautomeric forms, of which two predominant forms are 1,3-diketo form and 1,3-dienol form (Figure 1E). Although in the solid phase or solution the enol form is more stable, their relative concentrations may vary with temperature, polarity of solvent, pH, and substitution of the aromatic rings [16,17].

**Figure 2:**
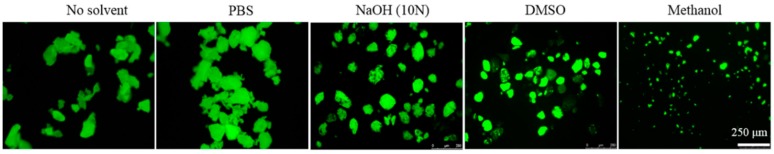
Curcumin solubility in different solvents. Please note that Cur is more soluble in methanol than in phosphate buffer saline (PBS), NaOH or dimethyl-sulfoxide (DMSO).

The amounts of keto-enol forms in Cur also play vital roles in the physicochemical properties, biological functions, and anti-oxidant activities [18]. The keto form is predominant in acidic (pH 3) to neutral conditions, while, the enol form is predominant in alkaline solutions (pH > 8), and is a potent free radical-scavenger [15]. Cur is hydrophobic in nature, so has poor solubility in water or hydrophilic solutions, although the solubility can be improved in basic conditions. Cur shows greater solubility in organic solvents (Figure 2), such as ethanol, methanol, isopropanol, acetone and dimethyl-sulfoxide (DMSO), whereas it is moderately soluble in hexane, cyclohexane, tetrahydrofuran and dioxane [15].

Interestingly, Cur is a natural fluorophore, with its absorption noted in polar solvents ranging from 408 to 540 nm [16,19]. The maximal fluorescence intensities of Cur is noted in chloroform, acetonitrile, and in acetone to be in the range of 494 to 538 nm, whereas in alcohols and dimethyl formamide (DMF), the fluorescence spectra may shift from 536 to 560 nm [15]. In contrast, in non-polar solvents (e.g., benzene, hexane and cyclohexane), sharper peaks (~460 nm) are observed, because of the blue-shifting of its absorption spectra [15] (Table 1).

### 2.3. Cur Biosynthesis

Cur may be biosynthesized via two ways (Figure 1F). Phenylalanine is the precursor molecule, and the cinnamic acid is the first byproduct of the Cur biosynthetic pathway (Figure 1F). When cinnamic acid reacts with 5-malonyl CoA, it forms bis-dehydroxybisdesmethoxy-Cur. This compound can then be converted to bisdemethoxy-Cur (BDMC) and demethoxy-Cur (DMC), which can be transformed into Cur (Figure 1F). The second pathway involved in Cur synthesis is the production of cinnamic acid, which is then converted to p-coumaric acid, and ferulic acid. The ferulic acid reacts with 5-malonyl CoA to form Cur [20,21] (Figure 1F).

### 2.4. Cur Metabolism

The metabolism of Cur, including its pharmacokinetics (PK) and pharmacodynamics (PD) has been studied by several investigators in rodents and in human [22,23,24,25]. The profile of Cur metabolites depends on the route of administration. For example, oral administration of Cur immediately reaches the liver, following intestinal absorption, and become sulfated or glucuronidated by liver-specific enzymes, such as sulfatase and glucuronidase, respectively (Figure 1). The major Cur metabolites in animal liver are the glucuronides of tetrahydrocurcumin (THC) and hexahydrocurcumin (HHC), whereas the traces amounts of dihydroferulic acid and ferulic acid are also found as the minor metabolites [26]. However, both these glucuronides, and sulfate conjugates are water soluble, and found in the urine of rats. According to Pan and colleagues, 99% of Cur in plasma was present as glucuronide-conjugates, which suggests that Cur first undergoes extensive reduction by alcohol dehydrogenase, followed by conjugation [27]. In contrast, when Cur is administered intravenously (i.v.), or intraperitonally (i.p.), it can form more stable, and water soluble Cur-derivatives, such as THC, HHC and octahydrocurcumin (OHC), which are easily eliminated from body through urine [27]. In addition, after absorption, Cur is readily catabolized to several degradation products, such as ferulic aldehyde, ferulic acid, ferulyol methane and vanillin (Figure 1G). A pharmacokinetic (PK) study revealed that the maximum concentration of curcuminoid conjugates in plasma was found within 1 h after its oral administration [28], but whether these Cur-metabolites are active, in a manner similar to free Cur, is not yet clear. However, some experimental data demonstrated that Cur-glucuronides and THC are less active than Cur itself [26], but other studies reported that they may be more active than Cur, because of their greater stability in body fluids, [29]. For example, THC shows better anti-diabetic and anti-oxidative effects than Cur in a rat model of type-2 diabetic [30], whereas Sandur and colleagues reported that THC has much lower anti-inflammatory and anti-proliferative activities than Cur [31].

## 3. Pleotropic Actions of Cur on Nervous System

### 3.1. Anti-Amyloid Properties

The most promising application of Cur in neurodegenerative diseases therapy is its anti-amyloid property [9,32]. Its preferential binding and potent inhibitory effects on amyloid aggregation has attracted researchers to investigate its beneficial roles for treating neurological diseases [13,33]. It not only binds with Aβ-oligomers and fibrils in AD [9,34], but also binds readily with other amyloid proteins, such as α-synuclein (α-syn) in PD [35], huntingtin (HTT) in HD [36], phosphorylated tau (p-tau) in tauopathies and AD [37], as well as with prion proteins in prion diseases [38] (Table 2). Most interestingly, the high lipid content of brain tissue allows lipophilic Cur molecules to cross the blood brain barrier (BBB) and inhibit the aggregation of amyloid proteins.

### 3.2. Potent Antioxidant

**Figure 3:**
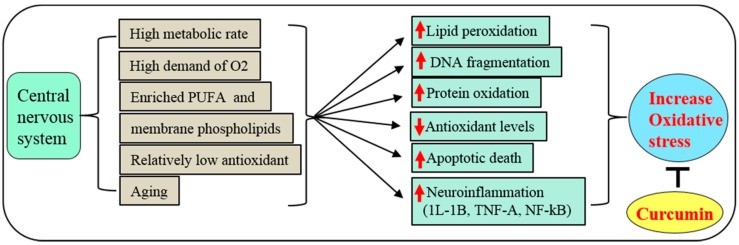
Amelioration of oxidative stress by Cur in brain. The CNS is vulnerable to oxidative stress due to high metabolic rate, which causes higher O_2_ demand. This leads to an increase in oxidative stress in the brain tissue. Whereas Cur, as a potent free radical scavenger, can ameliorate these effects.

Due to the high metabolic rate, increased demand of O_2_, large quantities of membrane phospholipids and polyunsaturated fatty acids (PUFA), and lower levels of anti-oxidants relative to other organs, the CNS is particularly vulnerable to oxidative damage (Figure 3). All these factors significantly contribute to increase reactive oxygen species (ROS) and peroxynitrite (ONOO-) levels, which lead to inflammation, mitochondria dysfunction and, ultimately, induce neuronal death. Chronic progressive neurological diseases induce inflammation, oxidative stress, lipid peroxidation, DNA damage, oxidized protein products [42].

Chronic oxidative stress has also been associated with induction of misfolded protein aggregates in brain tissues [43]. To counter this, Cur, as a potent anti-oxidant, can scavenge superoxide anions (O_2_^−^) and hydroxyl radicals (OH^−^), and increase anti-oxidant levels, such as glutathione (GSH) [44]. Cur also can stabilize the brain anti-oxidant enzyme systems, including activation of superoxide dismutase (SOD), glutathione peroxidase (GPx), glutathione S-transferase (GST) [45]. It also protects cells from lipid peroxidation, DNA damage, and protein oxidation or protein carbonylation [46] (Figure 3). Although most researchers describe oxidative stress as an imbalance of pro-oxidants and antioxidants, the actual mechanism involves a disruption of redox signaling and control [47,48]. Therefore, measurement of the signaling proteins associated with oxidative stress, as well as the ROS and anti-oxidant system, are required to investigate the effectiveness of Cur treatments.

### 3.3. Anti-Inflammatory Agent

**Figure 4:**
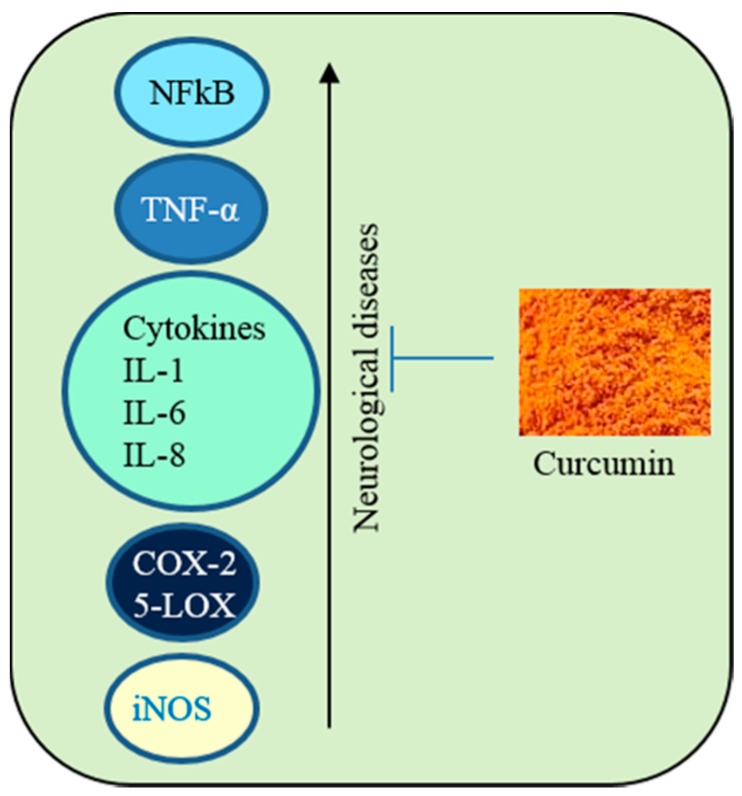
Anti-inflammatory properties of Cur. Curcumin increase levels of anti-inflammatory cytokines, inhibits inflammatory chemokines, iNOS levels and inhibits transcription factors, such as NF-κB.

Next to its anti-oxidant properties, the second most important reason for interest in Cur as a therapy for neurological diseases is its ability to reduce neuroinflammation [33,49]. Several reports suggest that Cur is a potent anti-inflammatory agent, which can downregulate many neuroinflammatory marker proteins, such as nuclear factor kappa beta (NF-κB) [45]. Cur also inhibits phospholipases and arachidonic acid metabolic enzymes, such as cyclooxygenase-2 (COX-2), 5-lipoxygenase (5-LOX) [50]. In addition, it reduces the levels of several cytokines, such as tumor necrosis factor (TNF), interleukin-1 (IL-1) and interleukin-6 (IL-6) [51,52] (Figure 4). Similarly, Cur is also an agonist for peroxisome proliferator-activated receptor gamma (PPARγ) which can inhibit the pro-inflammatory pathways [53].

### 3.4. Modulate Activity of Molecular Chaperones

Cellular protein quality is control by a set of proteins, called molecular chaperones. They are very important to reverse the unfolded or misfolded proteins to their native form. One of the important molecular chaperones are the heat shock proteins (HSPs). These proteins are downregulated in different neurological diseases [54]. Recently, we, and others, have shown that Cur is neuroprotective through the activation of molecular chaperones, such as HSP70, HSP90, HSP60 and HSP40 and heat shock cognate 70 (HSC70) [55].

### 3.5. Increase Neurotrophins, Neurogenesis and Synaptogenesis

**Figure 5:**
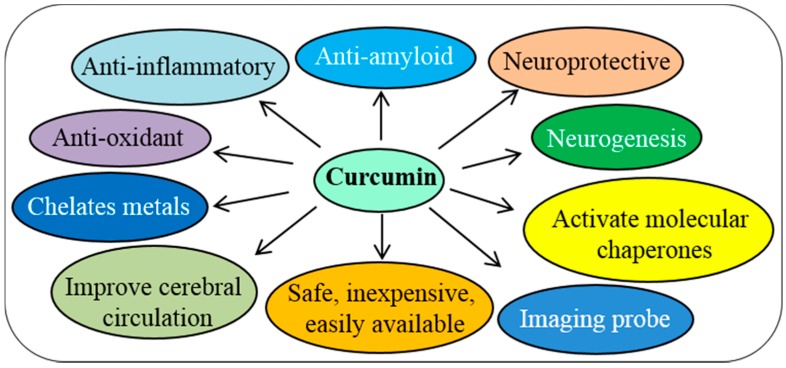
Uses and advantages of Cur for diagnosing and treating neurological diseases. In addition to its pleotropic therapeutic effects Cur is safe, inexpensive, and readily available and can be used to label Aβ deposits in the brain.

Mounting evidence indicates that significant declines in neuronal growth factors, such as NGF, BDNF, GDNF, as well as other supporting factors, such as PDGF can lead to synaptic damage and neuronal death. Diets containing Cur have shown to stimulate NGF, BDNF, GDNF, PDGF levels in vivo [56]. Cur also enhances neurogenesis, synaptogenesis and improves cognition in rats [57], which may be due to promoting these neurotrophic factors. Furthermore, improved memory functions in animal models of neurological diseases have been observed after Cur therapy, which may be due to increase levels of these neuronal growth factors. The pre-synaptic and post-synaptic markers, such as synaptophysin and PSD95, are also restored in different animal models of neurodegenerative diseases after Cur treatment [58] (Figure 5).

### 3.6. Metal Chelator

Heavy metals, such as aluminum (Al), copper (Cu), cadmium (Cd), iron (Fe), lead (Pb), manganese (Mn), and zinc (Zn) can induce misfolded protein aggregation and production of ROS in different neurological diseases [59,60]. The presence of two phenolic (OH) groups (see Figure 1) and one active methylene (CH2) group in a Cur makes it an excellent ligand for any metal chelation [61]. For example, Cur can interact with Cd and Pb and prevent neurotoxicity caused by these metals [62]. In fact, Cur effectively binds to Cu, Fe, and Zn, and makes them unavailable to induce amyloid protein aggregation. In addition, most of these heavy metals can induce neuroinflammation by increasing the expression of NF-κB levels, whereas it is speculated that Cur suppresses inflammation by inhibiting the NF-κB levels, perhaps via metal chelation [7,63]. The Cur-metal complexes also show greater anti-oxidant properties. For example, Cu-Cur complexes scavenge ROS more efficiently than Cur alone [61]. Similarly, Mn-Cur complex exhibits a more potent neuroprotection than Cur, as shown in both *in* *vitro* and in vivo experiments [18,61] (Figure 5).

### 3.7. Cur Regulates Epigenetics

Epigenetics play vital roles in gene expression in different disease conditions. Cur plays significant regulatory roles in modulating the methylation, acetylation, ubiquitination, and phosphorylation status of histone and other DNA-binding proteins [64]. For example, when the lysine at position 4 in histone-3 becomes methylated (H3K4me3), it activates the gene, whereas the lysine methylation at position 27 in the same protein (H3K27me3) silences the gene [65]. Similarly, histone acetylation produces increase in gene expression, whereas deacetylation has opposite effect. The histone acetylation is governed by histone acetyltransferase (HAT) and the enzyme involved deacetylation is the histone deacetylase (HDAC).

**Figure 6:**
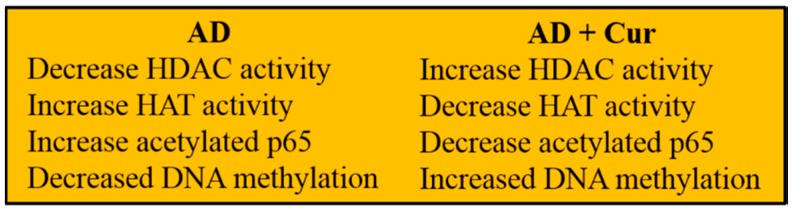
Effects of Cur on epigenetics in AD. Cur restores the activity of HDAC and inhibits HAT activity, along with increase DNA methylation in animal models of AD.

However, the epigenetic role of gene expression of Cur has been shown by inhibiting HAT activity and activating HDAC in AD (Figure 6)*.* Cur can directly bind to HAT at a nM levels and can inhibit the catalytic activity of HATs [64], thus inhibiting nuclear histone acetylation. Decreases in histone acetylation reduce the inflammation via NF-κB pathway in some brain diseases [45].

### 3.8. Improving Cerebral Circulation

Decreased cerebral circulation in aging brain causes an increase in risk of cerebral hemorrhage and stroke, whereas Cur has an influential role on cerebral circulation [66]. It can reduce the adhesion of platelets in brain microvascular endothelial cells (BMECs) [67,68], and also can inhibit the inflammation of blood vessels, which may improve overall cerebral circulation [69].

## 4. Limitations of Cur Delivery

Cur has been delivered in animals and humans by several means, including oral, subcutaneous, intraperitoneal, intravenous, nasal, and topical deliveries to achieve its beneficial effects. The major concerns about Cur delivery involve its instability and poor solubility in most body fluids, which reduces its absorption through the gastrointestinal (GI) tract, and facilitates its metabolism and degradation, as well as rapid elimination from the body, mitigating its bioavailability [70]. For example, researchers were unable to detect free Cur from the plasma of AD patient in a clinical trial in which 2–4 g Cur were delivered daily [71]. It is hypothesized that after absorption, Cur becomes rapidly glucuronidated in the liver by glucuronidase, which makes it water soluble, and, thus, promotes its rapid excretion through the urine [71,72]. Similarly, oral administration of 1 g/kg dose of Cur causes excretion of ~75% of Cur through feces, with negligible amounts in the urine [73]. Similarly, approximately 40% of the Cur was found in the feces, along with Cur-glucuronide and sulfates in the urine when 400 mg per day Cur is administered orally [26]. Most of the Cur is excreted within 72 h when lower doses (10 or 80 mg) are administered, whereas Cur remains in tissues for 12 days after oral administration of higher (400 mg) doses [24]. In contrast, no Cur or its metabolites were found in urine in a clinical trial when 36 and 180 mg was given daily for 4 months by oral administration, but some of these metabolites was excreted in the feces [74]. Clearly, major challenges for successful Cur delivery and its clinical applications for neurological diseases will require a special formula, which can optimize its solubility, stability, and bioavailability. In addition, it is critical to determine the amount of Cur required to prevent further neurodegeneration or to rescue degenerating neurons in neurological diseases.

## 5. Nano-Technological Approaches for Cur Delivery

To improve the bioavailability of Cur, numerous approaches and many promising novel formulations have been undertaken by several investigators, which included the use of nanoparticles, liposomes, micelles, and phospholipid complexes, nanogels, noisomes, cyclodextrins, dendrimers, silver, gold, and structural analogues of Cur [11] (Table 3). Most of these novel delivery mechanisms increase Cur bioavailability by providing longer circulation, better permeability, and/or resistance to metabolic processes.

### 5.1. Adjuvants

**Figure 7:**
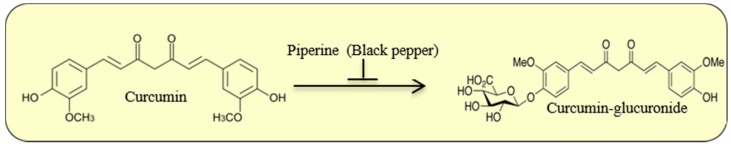
Use of adjuvant piperine to increase bioavailability of free Cur levels. Rapid glucuronidation process reduces bioavailability of Cur, whereas piperine present in black pepper can inhibit this glucuronidation process, thus increasing the amount of free Cur in different tissues.

Conjugation of piperine (extracted from black pepper, a well-known inhibitor of hepatic and intestinal glucuronidation of Cur) with Cur increase free Cur levels in animals and human plasma [76]. For example, co-supplementation with 20 mg of piperine with 2 g of Cur significantly increased the bio-availability of Cur by 2000 folds in a clinical trial [75] (Figure 7).

### 5.2. Bio-Conjugates

Several bio-conjugates (e.g., turmeric oil, glycine, alanine, and/or piperic acid) can be used to increase the cellular uptake and greater bioavailability of Cur [26] (Table 3). For example, when curcuminoid is combined with turmeric oil (turmerons) in a specific proportion to make Biocurcumax-95 (BCM-95), the Cur-bioavailability was 7-8 times more than that of natural Cur [77] (Table 4).

This formula has better absorption into blood and longer retention time compared to natural Cur. It also increases the activity of Cur up to a 700%, as confirmed by clinical trials [77]. Similarly, when Cur was bio-conjugated with glycine, alanine, and/or piperic acid, these formulae improved the anti-microbial properties over natural Cur, which suggests that these formulae promote cellular uptake, reduced their degradation, and increased Cur activity inside the cells [78]. In another study, when epigallocatechin-3-gallate (EGCG, a polyphenol of green tea) was conjugated with Cur, this formula also increased Cur uptake, as well as its beneficial effects [79].

### 5.3. Cur-Phospholipid Complex

Cur-phospholipid complex increased aqueous solubility of Cur up to 3-fold and showed greater protection against tissue damage compared to unformulated Cur [80]. Oral administration of Cur–phospholipid complex (100 mg/kg) in rats showed a maximum plasma Cur levels of 600 ng/mL after 2.33 h, whereas administration of the same amount (100 mg/kg) of unformulated Cur, (i.e., the free Cur) was ~267 ng/mL after 1.62 h, indicating that the Cur-phospholipid complex has greater bioavailability than unformulated Cur. Importantly, this formula provides more tissue protection by increasing antioxidant enzyme systems. Similarly, a formula of phosphatidylcholine with Cur, when administered orally (340 mg/kg) in rat, showed an increase in Cur-bioavailability by 5-folds in comparison to unformulated Cur [80].

### 5.4. Liposomes

Liposomes are spherical, self-assembling, closed colloidal structures, composed of lipid bilayers, with both hydrophilic and hydrophobic characteristics (Table 5). Therefore, they provide an excellent system for delivering hydrophobic compounds, such as Cur. Liposome vesicles are typically 25 nm to 2.5 mm in diameter and can be extracted from natural phospholipids or can be artificially synthesized. Cur can be encapsulated with liposomes, which can be delivered into the cell by membrane fusion or endocytosis, a formulation which has proved to be safe and to enhance Cur solubility and cellular activities. Moreover, liposome-encapsulated Cur is transported without rapid degradation, along with minimum side-effects, and with greater stability. In addition, liposomal-Cur is more stable than free Cur in PBS, but both are equally stable in human plasma and in culture media. Furthermore, the liposome-Cur complex increases the bioavailability and efficacy of Cur after intravenous administration in animals. This formula also possesses anti-cancer effects, both in vitro and in vivo, against osteosarcoma and breast cancer, and inhibits the growth of melanoma cells. It also increases serum creatinine, while decreasing tissue damage, cell death, and inflammation [11].

### 5.5. Micelles

Unlike liposomes, micelles are monolayered phospholipid complexes (in solution) with various shapes (spherical, vesicles, rod-like, or star-shaped) and sizes. They are amphipathic molecules, form emulsions when and solubilized in water, and act as excellent surfactants. A major advantage of using micelles for Cur delivery is that they are of smaller sizes (~10–100 nm in diameter), making them more stable in biological fluids [81]. Micelles can form a nano-sized core/shell structures in aqueous media, facilitate the permeability of hydrophobic drugs, such as Cur, by burring themselves inside the hydrophobic core and making the polymer more water soluble. Thus, micelles can act as transporters of Cur, and increase their efficiency by targeting specific organs, such as the brain. Using a solid dispersion method, Liu and colleagues [72] prepared bio-degradable, self-assembled polymeric micelles, loaded with Cur, which significantly increased the release of free Cur. In vitro results of studies using spherical Cur-loaded mixed micelles revealed an enhanced solubility and biological activity of Cur [82]. Similarly, 𝜀-poly-lysine micelles coated with curcuminoid also improved their solubility and cellular anti-oxidative activities, in comparison to free curcuminoid [83]. Furthermore, novel biodegradable micelles that were synthesized by conjugating methoxy-polyethylene glycol provided sustained Cur release for 24 h in vitro and enhanced its aqueous solubility and stability with a 3-fold reduction in IC_50_ value of Cur [84]. To increase prolongation of its half-life, higher bio-distribution, and bioavailability, while decreasing total clearance of Cur, Song and colleagues [85] synthesized a poly (d, l-lactide-co-glycolide)-b-poly(ethyleneglycol)-b-poly(d, l-lactide-coglycolide) (PLGA-PEG-PLGA) polymeric micelles which coated Cur. This prolonged its circulation time because of its smaller size and hydrophilic shell that reduced the drug uptake by the mononuclear phagocytic systems [85]. In an attempt to increase the aqueous solubility of hydrophobic drugs, a polymeric micellar formulation containing methoxy poly (ethylene glycol)-block-polycaprolactone diblock copolymers (MePEG-b-PCL) provided a 13 to 105 fold increase in solubility [11]. However, Cur-loaded micelles can boost the efficiency of the drugs by targeting specific cells, resulting in less drug accumulation in healthy tissues and a reduction in toxicity. Therefore, micelle encapsulation of Cur provides an enormous increase in solubility and bioavailability of Cur, making this formulation a very promising avenue for developing clinically effective therapeutic tools.

### 5.6. Noisome

Chemically, noisomes are alkyl or dialkyl polyglycerols that contain cholesterol, which acts as nonionic surfactant [86]. It is an excellent carrier for all kinds of drug molecules, including those which are hydrophilic, amphiphilic, or lipophilic in nature. Noisomes behave similar to liposomes in vivo, therefore, providing an alternative for liposome-based drug delivery. In addition, they have significant potential for anti-cancer and anti-inflammatory activity [81]. Because, they are very stable, which prolongs their delivery and suppresses the level of degradation, their use can improve oral bioavailability of Cur and can increase its skin penetration [87]. Therefore, noisomes are a potential delivery system for Cur that would increase its stability and bioavailability.

### 5.7. Nanogels

These are covalently cross-linked polymers with 3-D chain networks making them suitable for delivering bio-compatible drugs to different tissues (Table 5). They provide the perfect reservoir for loading and delivering different amphipathic drugs and prevent them from environmental degradation [88]. The size of nanogels can be customized by manipulating the functional groups used, the density or degree of cross-linking, ionic strength, and pH of the solution [88]. Several chemical interactions, such as salt bonds, hydrogen bonds, or hydrophobic interactions can be used to react with Cur to enhance its stability in biological fluids, as well as its oral and brain bioavailability [89]. For example, a self-assembled dextrin nanogel was used for Cur delivery, which proved to be a suitable carrier for controlling the release of Cur. By using dynamic light scattering (DLS), scanning electron microscope (SEM), and Fourier transform infrared spectroscopy (FTIR), it was shown that Cur-chitin nanogels had higher levels of Cur release at an acidic pH compared to neutral pH, and proved to be a potent toxic agent to cancer cells (0.1–1.0 mg/mL), without harming normal cells [90]. Furthermore, water-dispersible hybrid nanogels have been made by coating silver or gold bimetallic nanoparticles for intracellular delivery of Cur. This formula increases the Cur loading yields and its sustained release, along with its bioavailability, and also prevents Cur from surrounding temperature or exogenous irradiation with near-infrared light [91]. Therefore, nanogels might be an excellent carrier for Cur, due to their smaller particle size (10–200 nm), which significantly enhances their biodegradability, stability, loading efficiency, and/or biocompatibility, while prolonging half-life, increasing transdermal penetration and providing protection against degradation by the immune system [88]. Overall, the use of specifically designed, multifunctional hybrid nanogels appears to be safe and appropriate for Cur delivery in clinical trials aimed at the prevention of neurodegeneration or cancers.

### 5.8. Chitosan

This is a linear polysaccharide of deacetylated and acetylated units of chitin, present in exoskeleton of crustaceans and the walls of the fungi cells. They contain primary amine groups, which make it cationic in nature and increase its solubility in various media, while possessing its polyelectrolyte behavior, metal chelation, and structural uniqueness [92]. Chitosan-coated nanocarriers, contain Cur particles, and are positive in nature, are 114–125 nm in diameter, and can increase the fluorescence intensity of Cur. Cur-phytosome-loaded chitosan microspheres also improve Cur absorption, prolong the retention of Cur and increase its bioavailability due to the accumulation of nanoparticles in the ER [93]. Furthermore, binding of Cur to chitosan nanoparticles improves its chemical stability and prevents its degradation. Overall, Cur-coated chitosan derivatives can easily enter the cell membrane and release Cur in a controlled manner, and are nontoxic to normal cells, while toxic to tumor cells, at the same time maintaining stronger antioxidant and chelating effects than free Cur [94].

### 5.9. Gold Particles

Due to their optical and electrochemical uniqueness, stability in biological systems, capability for combining with biomolecules, and their lower cytotoxicity, gold nanoparticles (AuNPs) can be a potent carrier for Cur [95]. These particles can also be easily synthesized and functionalized and have improved longevity in the circulatory system. A formulation of chitosan-Cur nano-capsules with AuNPs formulated by a solvent evaporation method produced 18–20 nm diameter of AuNPs-Cur, which provides a more controlled and steady release of Cur, compared with Cur-encapsulated chitosan nanoparticles. The effect of Cur-conjugated-AuNPs on peripheral blood lymphocytes are those typical characteristics of apoptosis, including chromatin condensation, membrane blebbing, and the occurrence of apoptotic bodies [96]. Therefore, Cur conjugated AuNPs could provide better targeting of cells, sustained release of Cur, and more powerful antioxidant effects than free Cur.

### 5.10. Silver Particles

As a safe and potent anti-microbial agent, silver (Ag) can be used to improve Cur delivery [97]. Using a diffusion mechanism, researchers loaded Cur into a 15-nm diameter sodium carboxyl methyl cellulose silver nanocomposite (AgNPs) film. This film improved Cur encapsulation and increased its anti-microbial activity. Similarly, a novel hydrogel-AgNPs-Cur composite has been developed which produces greater anti-microbial activity than AgNPs-Cur films that lack the hydrogel. Moreover, there is a sustained release of Cur from the Ag-encapsulated composite, which can increase its bioavailability, as well as therapeutic values [11]. In addition, the use of AgNPs could protect the cells from anti-microbial attack and also act as an anti-inflammatory, anti-viral, and anti-cancer agent, along with its wound-healing properties [11] (Table 5).

### 5.11. Cyclodextrin

**Figure 8:**
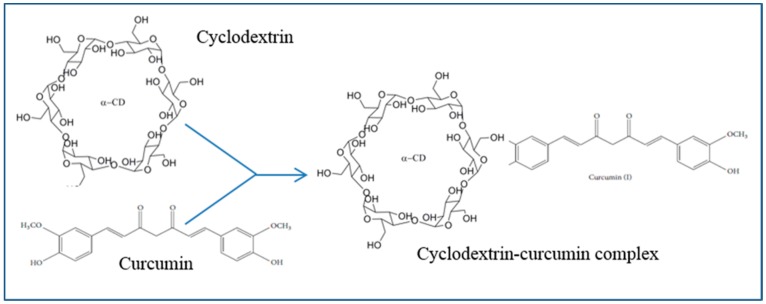
Cyclodextrin-Cur complex. Cyclodextrin is a water-soluble pseudo-amphiphilic starch molecule, which is a potent carrier for Cur and can increase its solubility and bioavailability.

Cyclodextrins (Cds) are the cyclic oligomers of glucose or oligosaccharide residues synthesized from starch molecules, which have wide applications, including use in pharmaceutical, drug-delivery, and food processing industries [81]. Chemically, they are pseudo-amphiphilic molecules, which help their solubility and stability in aqueous solution and can act as vehicles for oral or intravenous delivery of hydrophobic molecules (e.g., Cur) to improve their bioavailability and prevent their degradation without alteration of their pharmacokinetics [81]. A preparation with Cds-Cur complexes (Figure 8) improved the hydrolytic stability of Cur with enhancement of photodecomposition efficiency in organic solvents, thus increasing their stability and reducing their degradation rate compared to the free Cur [98].

Similarly, Cur tagged with β-Cds nanosponges has more solubilization efficiency and can release Cur more readily, given that the complex is nonhemolytic in comparison to free Cur [99]. Another study showed that the Cds-Cur complex becomes more potent as an anti-inflammatory agent than free Cur by inhibiting the nuclear factor kappa beta (NF-κB), inducing death of cancer cells [100], such as prostate cancer cells, while acting as a telomerase inhibitor. Therefore, Cds could encapsulate Cur and increase its stability and bioavailability, compared to the free Cur, without altering their pharmacokinetics.

### 5.12. Dendrimer

**Figure 9:**
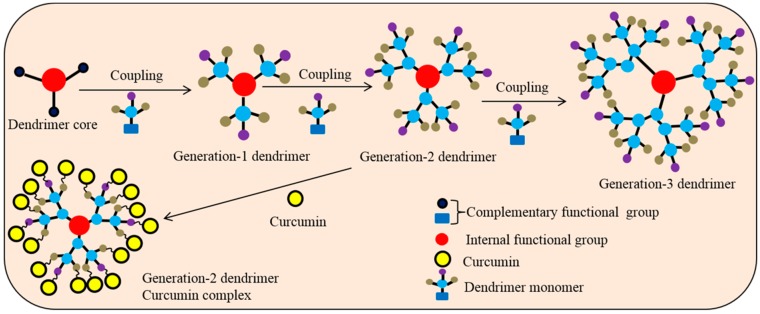
Schematic diagram showing dendrimer structure and construction of dendrimer-curcumin complexes. The Cur can be coated with the outer surface of the branched dendrimer, thus it can carry numerous Cur molecules, depending on the surface groups and charge.

Dendrimers are a group of small (nM), dense spherical, branched series of polymeric globular polymers (Figure 9), which are considered as synthetic proteins. They consist of a core, branched interiors and numerous surface functional groups (i.e., OH or NH_2_), which serve as a platform for carrying and delivering many drugs, (like Cur), small molecules, or DNA [101]. As a safe molecule, dendrimers can be used as a probe for molecular imaging. Dendrimer-Cur formulations are readily dissolved in aqueous solution, can increase cellular uptake, and show more cytotoxic effects in human breast cancer cells than free Cur. The dendrimer surface containing poly-amidoamine group can carry hydrophobic drugs (such as Cur) for their successful delivery. For example, a poly-amidoamine (PAMAM) encapsulated Cur conjugates show significant inhibition of telomerase activity and induce apoptosis by enhancing Cur uptake in human cancer cell lines [102].

Furthermore, when Cur is conjugated with dendrosomal nanoparticles, which are neutral, amphipathic, and biodegradable nanomaterials, this compound increases the stability and, uptake of Cur, while increasing its antitumor activity through its induction of apoptosis, as demonstrated in both in vitro and in vivo experiments [103]. In addition, the dendrosomal nanoparticle-Cur has chemo-protective and chemotherapeutic effects on colon cancer by inhibiting the cell proliferation and induction of apoptosis [103]. These properties make dendrimers especially attractive as a carrier for Cur, relative to other nanoparticles.

### 5.13. Solid Lipid Nanoparticles (SLNP)

**Figure 10:**
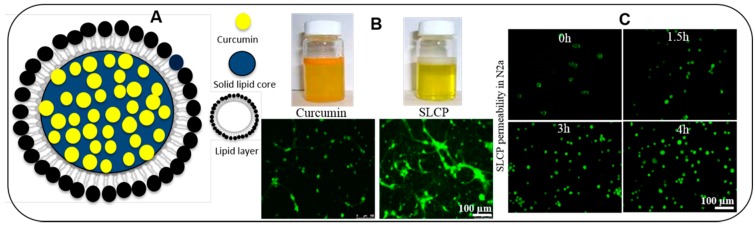
Schematic diagram showing formulation of solid lipid Cur particle (SLCP). (**A**) In this formula, outer layer is composed of long chain fatty acid bilayers, with the inner layer being composed of a solid fatty acid core and on that core that is coated with Cur molecules. (**B**) Comparative solubility (**upper**) and cellular permeability in primary hippocampal neurons (**lower**) and (**C**) permeability of Cur and SLCP in N2a cells. Scale bar = 100 µm

SLNPs are the spherical and submicron colloidal lipid carriers (50 to 1000 nm) which maintain a solid shape at room temperature (Figure 10). There are several advantages for using of SLNPs for Cur delivery, including improvement of release kinetics, enhancement of bioavailability, increased protection via encapsulation, ease of manufacturing, increased stability, along with versatile applications [104]. Moreover, the size of the Cur-SLNPs is much smaller, ranging from 100 to 300 nm, with a very favorable total drug content of <92% when manufactured by micro-emulsification technique. One of our recent experiments with SLNPs-Cur, using a dose of 555 ppm, showed that Cur level was 250–300 nM in mouse brain tissue, along with improved neurobehavioral outcomes [12,40].

In an experimental set up using an animal model of cerebral ischemia, rats fed with SLNPs-Cur had a 90% improvement in cognitive function, along with a 52% inhibition of acetyl cholinesterase activity [105]. Furthermore, this formula has been shown to increase the levels of superoxide dismutase (SOD), catalase, glutathione (GSH), and the activities of mitochondrial enzymes, while decreasing lipid peroxidation and peroxynitrite levels. This formula also improved the bioavailability of Cur in the brain by 16.4- and 30-fold with oral and intravenous administration, respectively. Similarly, solid lipid microparticles of Cur that were prepared with palmitic acid, stearic acid, and soya lecithin, had more powerful anti-angiogenic and anti-inflammatory activities. As such, the SLNP-Cur formula has several advantages over other nanoparticles, such as: (a) larger carrying capacity of Cur; (b) ease of scaling and sterilization; (c) protection via encapsulation, (d) more favorable kinetics, (e) increased bioavailability, (f) ease of manufacturing, and (g) superior stability with application versatility [11]. For example, Verdure Sciences has developed a SLNP-formulation of Cur, called “Longvida”, which achieves a 0.1 to 0.2 µM plasma level with an associated 1–2 µM brain level of free Cur in animals [6,10,13,55]. Later they optimized this formula as “lipidated Cur” which can achieve more than 5 μM in the brains of mice [106,107]. We have been working with this formula and found significant beneficial effects both in vivo and in vitro models of AD [108], and in an in vitro model of glioblastoma [14].

### 5.14. Derivatives and Analogues of Cur

**Figure 11:**
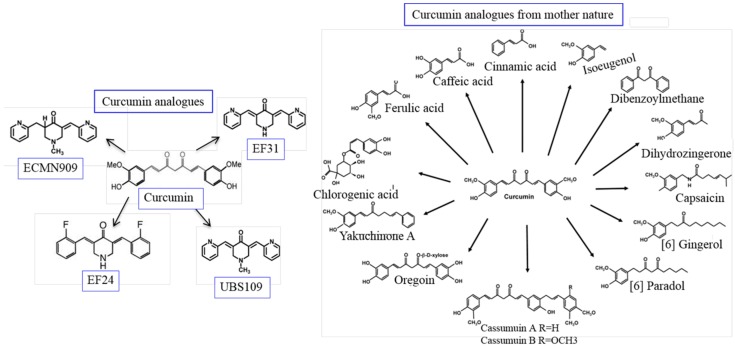
Different Cur analogues and derivatives. By modifying the structure, several analogues and derivative of Cur have been developed by many researchers, which improved its solubility, stability, bio-availability, and biological activities.

The biological properties of Cur and its derivatives depend on the chemical structure of Cur. For example, isomeric forms of Cur have better antioxidant properties. Therefore, structural modifications of Cur might be a good strategy to improve its biological activities. Several Cur derivatives and/or analogues have been synthesized and tested by many researchers. Among them, EF-24, a Cur analogue (Figure 11) has shown to possess promising anti-tumor activity in vitro and in vivo, in comparison to natural Cur [109]. Up to 32 mg/kg of this compound was safe in mice after intravenous administration, and the absorption was rapid after both oral and i.p. administration. At this dose, when mice were injected with EF-24 i.p., within 3 min, the peak plasma concentration of Cur reached 1000 nM and the absorption and elimination half-life values were 177 and 219 min, respectively. The bioavailability of oral and i.p. EF-24 was 60% and 35%, respectively [109].

These new analogues exhibit no in vivo toxicity and have shown growth suppressive activity that is ~30 times greater than that of natural Cur [110]. Furthermore, synthesized Cur analogues, when complexed with other chemicals, such as sodium dodecyl sulfate and cetyl-trimethyl-ammonium bromide micelles, show anti-oxidative effects against free-radical-induced lipid peroxidation [111], suggesting that synthesized Cur can be used as an antioxidant, as is the case with natural Cur.

## 6. Rationale for Cur Therapy in Neurodegenerative Diseases

**Figure 12:**
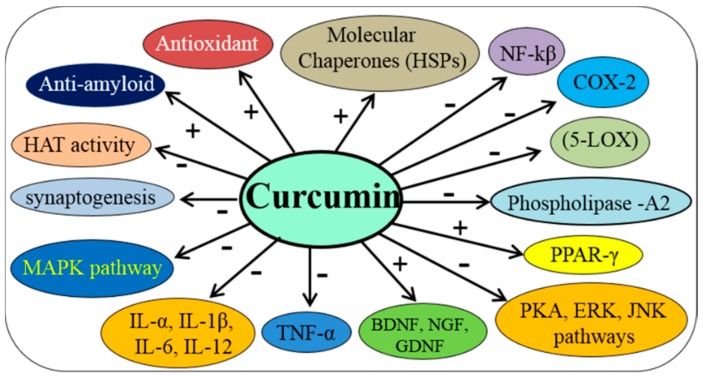
Multiple-reasons for use Cur for treating neurodegenerative diseases. Among these, its anti-amyloid property is particularly attractive as a therapeutic tool.

Several experiments have demonstrated that Cur has pleiotropic effects on the nervous system. It is a neuroprotective agent, with potent antioxidant properties, along with the significant anti-inflammatory activity [26,33]. Therefore, its anti-amyloid properties make it a most promising compound for treating different brain diseases caused by amyloid accumulation. In addition, Cur is hydrophobic, as well as lipophilic in nature, and because the brain contains huge amounts of lipids, the absorption, bioavailability, and half-life profiles of Cur are very favorable in the CNS. Several experiments have shown that neuroinflammation, oxidative damage, and deposition of misfolded amyloid proteins synergistically contribute to the pathogenesis of many neurological diseases. Therefore, targeting these processes is a prime strategy for developing therapies for different neurodegenerative diseases. In this context, use of Cur as a treatment for neurodegenerative diseases, has several advantages (Figure 12), including it can: (i) readily cross the blood brain barrier [13,32]; (ii) bind and dis-aggregate amyloid oligomers and fibrils (anti-amyloid) [9,112]; (iii) enhance amyloid clearance similar to vaccine [113]; (iv) reduce chronic inflammation in neurodegenerative diseases; (v) act as a potent antioxidant; (vi) stimulate neurogenesis, as shown in animal models; (vii) chelate metals, including removal of the metals from Aβ; (viii) be taken at relatively high doses (12 g/day) with no negative effects; (ix) be obtained readily and inexpensively; (x) be absorbed into hydrophobic and lipophilic nature, and (xi) produce high fluorescent intensity when it binds to amyloid-plaques, for use in labeling and imaging of amyloid plaques ex vivo and in vivo, or as an imaging probe for non-invasive techniques [13,114] (Figure 12).

### 6.1. Curcumin Therapy in Alzheimer’s Disease

Alzheimer’s disease is the major age-related neurodegenerative disease, characterized by various neurobehavioral abnormalities, but most prominently by early memory deficits, with a gradual decline of cognitive and intellectual functions that culminate in dementia. It is the leading cause of death in the elderly [115]. The hallmark pathologies of AD are the deposition of Aβ protein as senile plaques in extracellular spaces [116,117,118,119,120] and the phosphorylated tau as neurofibrillary tangles (NFT), intracellularly [121,122,123,124,125]. The accumulation of these abnormal or misfolded proteins are thought to be the principal reasons for the synaptic deficits, neuronal loss, oxidative damage and increase in neuroinflammation in numerous brain regions. Therefore, drugs with pleotropic actions, especially those with anti-amyloid properties and those that reduce oxidative damage, and neuroinflammation should provide the greatest potential for preventing the neuronal loss observed in AD. Unfortunately, numerous drugs or small molecules that have been developed and tested to halt neurodegeneration have not prevented or reduced the symptoms of this disease. Recently, Cur is being considered one of the most potent and promising natural polyphenols for use in AD therapy, due to its pleotropic actions (See Table 6).

#### 6.1.1. Inhibition of Aβ Aggregation

**Figure 13:**
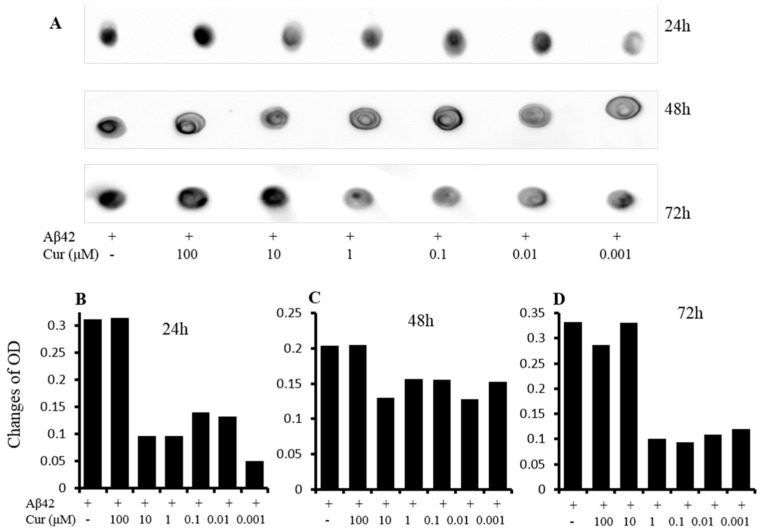
Nanomolar (nM) concentrations of Cur inhibit Aβ42 aggregation in vitro. HFIP-treated Aβ42 was incubated with and without different concentrations of Cur for 24–72 h and a dot blot was performed by probing with Aβ42-fibril specific antibody (OC) and the color was developed with chemiluminescent reagents and the optical density of each dot was measured using Image-J software. Lower concentrations (1–0.001 µM) of Cur inhibited Aβ42 aggregation, whereas higher concentrations had no effect on aggregation.

Numerous experiments have demonstrated that Cur can directly bind to the β-pleated sheet structures of Aβ. Interestingly, Cur shows the strongest inhibitory effects on Aβ aggregation among 214 antioxidant compounds tested in vitro [125,135,139,140], indicating it is one of the most potent anti-amyloid compounds investigated so-far. An in vitro study conducted by Ono and colleagues has demonstrated that Cur has a dose-dependent effects on the inhibition of Aβ1−40/1−42 fibrils, with an EC_50_ of 0.09–0.63 μM [9,112]. Several in vitro studies have demonstrated that Cur can attenuate the assembly of both Aβ40 and Aβ42 oligomers and fibril formation [7] (Figure 13).

Following oral or intraperitoneal injections of Cur for 3–7 days in mice, Cur crossed the blood-brain barrier (BBB) and was found in brain tissue, decreasing neuropathology in an animal model of AD (Table 7), as shown by two-photon microscopic imaging [141]. Similarly, significant inhibition of Aβ oligomerization, its plaque formation, and tau phosphorylation, along with behavioral improvements, were observed in a mouse model of AD after oral administration of Cur [9,33,51]. Furthermore, in vivo imaging, using multiphoton microscope, showed a decrease of 30% Aβ plaque size and prevented dystrophic neurites when the animals were injected the Cur via tail vein for one week [141]. In another study, Cur was shown to bind with Aβ-plaques in retina [6,25,139]. In a clinical study, Cur engulfed Aβ effectively and decreased plaque load in AD brain [9]. Though there are no true epidemiological studies that relate Cur intake to the incidence of AD, a trend for reduced incidences of AD is observed among Indian and South Asian countries, in which Cur is consumed everyday as a spice, when compared to the United States and other Western countries in which the intake of Cur is much less [33].

#### 6.1.2. Inhibition of Aβ Production

**Figure 14:**
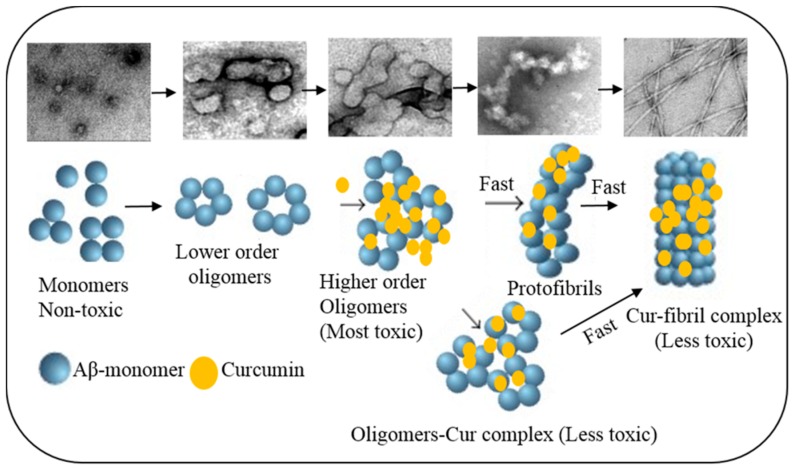
Schematic diagram showing the formation of different Aβ-species during its aggregation process and the inhibitory role of Cur in its assembly process. Cur has been shown to bind with Aβ and attenuate the oligomer formation or slowdown the process. Additionally, it can fasten the transformation of more toxic oligomers into less fibril forms.

Aβ is a by-product of a transmembrane protein, called amyloid precursor protein (APP). The production of Aβ is catalyzed by the two successive enzymes, first by β-secretase (BACE), followed by γ-secretase, which contains presenilin-1 (PS-1). It is speculated that during disease progression, induction of inflammatory signals aggravate the expression of Aβ production by increasing the activity of BACE [142], whereas Cur inhibits the activity of BACE, thus reducing the levels of Aβ [9,33]. In addition, Cur is a potent inhibitor of the APP metabolic pathway, thus lowering Aβ levels [7,143]. Furthermore, it can regulate Aβ production by inhibiting GSK-3β-mediated PS-1 activation [144] (Figure 14).

#### 6.1.3. Aβ Clearance

**Figure 15:**
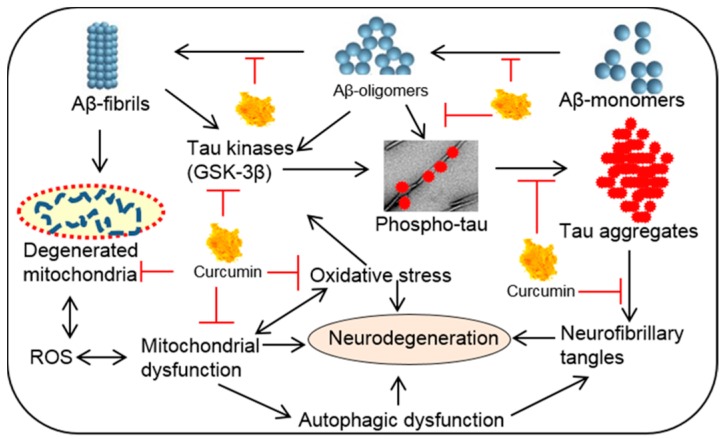
Schematic diagram showing the role of Cur in Aβ clearance. Cur stimulates phagocytosis of Aβ by activating microglia and enzyme-mediated degradation of Aβ. Cur also stimulates B-lymphocytes to activate Aβ-specific antibody, which neutralizes Aβ. In addition, it also inhibits Aβ-influx from the blood stream to the brain and increase Aβ-efflux from brain to the general circulation. “↔ “bidirectional; “→ “ increased; and “┬” inhibition/decrease.

The levels of Aβ in the brains of AD patients depend on a balance between production, clearance, and influx of Aβ. When clearance pathways are impaired the levels of Aβ are increased. However, there are several ways in which Aβ is disposed from the cell, including receptor-mediated Aβ transport across the BBB and enzyme-mediated Aβ degradation, as well as the involvement of immune system [145]. Cur can act in a manner that is similar to an amyloid vaccine [33] and can bind with Aβ to enable its removal from the brain by promoting receptor-mediated Aβ efflux [9]. In contrast, Cur could decrease Aβ load by suppressing the Aβ influx across the BBB and by upregulating the enzyme-mediated degradation of Aβ. Furthermore, Cur can stimulate phagocytosis and increase the association of phagocytic cells around Aβ-plaques as observed in a rat AD model [146] and the Tg2576 mouse model of AD, as well as with plaques in post-mortem human brain sections exposed to primary rodent microglia [147] (Figure 15).

#### 6.1.4. Inhibition of Tau Phosphorylation

The second most common pathology observed in AD is the tau tangle, which is basically the deposition of phosphorylated tau (pTau) as paired helical filaments (PHF). Tau is a microtubule stabilizing protein, which is abundant in neurons of whole CNS. Hyperphosphorylation of tau causes cytoarchitectural changes, which create oxidative stress, mitochondrial dysfunction, and neurodegeneration [148]. Tau phosphorylation and deposition of NFTs are regulated by several tau kinases, with glycogen synthase kinase-3β (GSK-3β) and mitogen-activated protein kinase (MAPK) being the most common among them [149]. The common tau-kinases, which can phosphorylate the tau protein are cyclin-dependent kinase 5 (Cdk5)/p25, extracellular signal-regulated kinase 2 (ERK2), S6 kinase (S6K), microtubule affinity-regulating kinase (MARK), SAD kinase (SADK), protein kinase A (PKA), calcium/calmodulin-dependent protein kinase II (CaMKII), or Src family kinases, such as Fyn and c-Abl.

**Figure 16:**
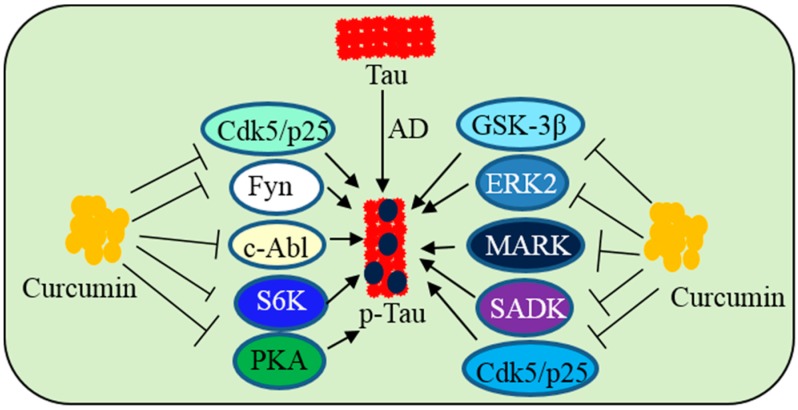
Schematic diagram showing the role of Cur in inhibition of tau phosphorylation in AD. Cur has been shown to inhibit tau-kinases, thus inhibiting phospho-tau formation. It also binds with tau directly to inhibit their aggregation.

Therefore, inhibition of tau kinases could be a viable strategy to prevent NFT-induced neurodegeneration. Cur has been shown to bind to NFTs in human AD brain and mouse models of AD [129] (Figure 16). An in vitro experiment showed that Cur inhibits pTau aggregation by reducing oxidative stress [150]. We have shown that Cur inhibits GSK-3β activity and reduces tau dimer and pTau oligomerization in a human tau transgenic mouse model [12]. In addition, oral administration of Cur (555 ppm) together with DHA, reduced pTau by inhibiting IRS-1 and JNK activities in vivo [12] (Figure 16).

#### 6.1.5. Inhibition of Oxidation and Inflammation

**Figure 17:**
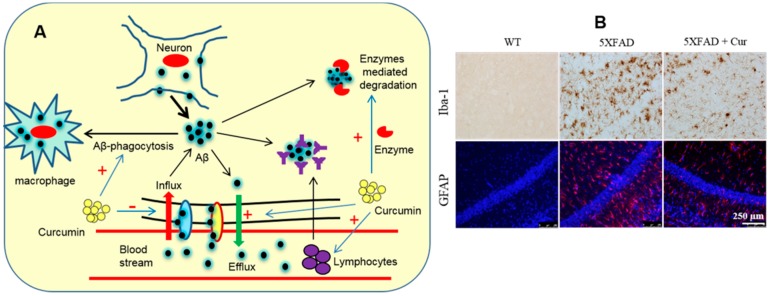
Schematic diagram showing the anti-inflammatory properties of Cur in Alzheimer’s disease. (**A**) Cur stimulates B-lymphocytes to produce antibodies, increase anti-inflammatory cytokines and decrease proinflammatory chemokines, increase phagocytosis of Aβ, and increase proteolytic enzymes to degrade Aβ. (**B**) Cur inhibits microglia (Iba-1; **upper**) and astrocyte (GFAP; **lower**) activation in 5xFAD mice brain tissue.

Whether or not Aβ can induce oxidative stress and neuroinflammation is not yet clear, but it is considered one of the primary events involved in neuronal death in AD [151]. However, as a strong antioxidant, Cur can limit the pro-oxidant, pro-inflammatory, and other toxic effects in AD brains [7,33]. Cur can inhibit the inflammatory cytokines, including IL1, IL6, TNF-α, IFN-γ, and COX-2 activity [152]. Several studies have demonstrated that Cur can inhibit activated astrocytes and microglia, as shown by reducing GFAP and Iba-1 levels [108,153] (Figure 17). Therefore, as an anti-inflammatory natural polyphenol, Cur is a promising compound for tackling oxidative stress and inflammation in AD.

#### 6.1.6. As an Imaging Probe for Aβ-Plaque Detection Ex Vivo and In Vivo

**Figure 18:**
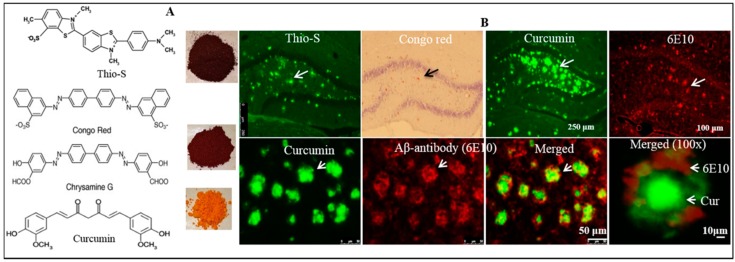
Cur binds to Aβ-plaques greater than classical amyloid binding dyes. (**A**) Curcumin has structural similarities with classical amyloid binding dyes. (**B**) **Upper panel**: Cryostat sections from 5XFAD mouse hippocampus were stained with Thio-S, Congo-red, Cur and Aβ-specific antibody (6E10). Please note that Cur stained Aβ plaques are more visible than other dyes. **Lower panel**: Cur is co-localized with Aβ-specific antibody (6E10) in Aβ-plaques in mouse cortical tissue from 5xFAD.

Cur is an ideal fluorophore for Aβ plaque imaging and detection because it is a natural fluorescent molecule and preferentially binds to Aβ plaques [6,9,13,25,141]. Therefore, researchers use Cur for labeling and imaging Aβ-plaques ex vivo and in vivo [13]. Interestingly, it has structural similarities with classical amyloid binding dyes, (such as Thioflavin-S, Congo red, and crysamine-G), which makes it a promising candidate for labeling and imaging of Aβ plaques ex vitro and in vivo [13] (Figure 18). For example, Garcia-Alloza and colleagues have demonstrated that Cur can be used to visualize Aβ-plaques in vivo, as shown in APP-tau transgenic mouse model [141]. Similarly, a strong fluorescent signal was observed when the brain sections both from animal models of AD and from AD patients were incubated with Cur [6,154]. To confirm whether Cur binds to Aβ-plaques, we performed immunohistochemistry in the 5XFAD brain tissue sections with Aβ-specific antibodies (6E10) and then the same sections were stained with Cur, and we observed that Cur was completely co-localized with Aβ-specific antibody (Figure 18), which indicates that Cur has specificity to Aβ similar to Aβ-specific antibody [13].

**Figure 19:**
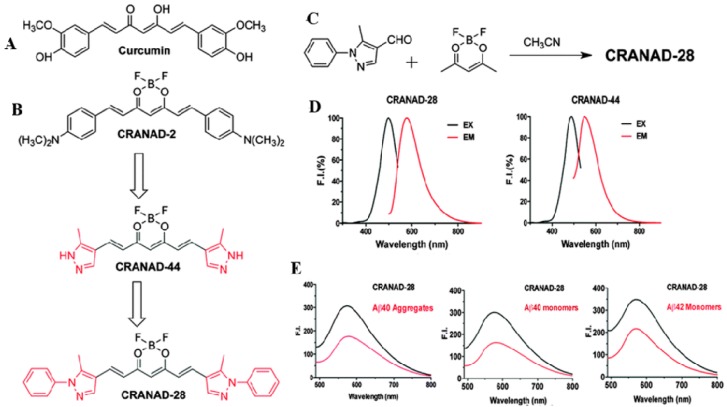
Structural modifications of Cur. (**A**) Structure of natural Cur; (**B**) Design of CRANAD-28 through pyrazole replacement; (**C**) The synthetic route for CRANAD-28 synthesis; (**D**) The excitation/emission spectra of CRANAD-28 and -44; (**E**) fluorescence responses of CRANAD-28 with Aβ40 aggregates, Aβ40 monomers and Aβ42 monomers.

More recently, researchers have tried to use the fluorescent properties of Cur derivatives for in vivo imaging, such as positron emission tomographic (PET) probes for amyloid imaging or retinal scan for detection of AD in experimental animals and humans [127]. However, it is not a practical probe for in vivo near infrared (NIR) imaging, due to its short emission wavelength (~550 nm), limited bioavailability, and rapid degradation. To overcome these issues, scientists modified the Cur structure to form boro-fluoro-Cur derivatives, which shift the emission wavelength to the NIR range (Figure 19). These derivatives are called CRANAD derivative (e.g., CRANAD-2, CRANAD-44 and CRANAD-28) [155] (Figure 19). These derivatives of the Cur probe significantly increase fluorescence properties upon binding to Aβ-plaques [155,156]. Surprisingly, the binding affinity of Cur for Aβ aggregates is higher (with a Ki of 0.07 nM) for F18-labeled Cur binding of fibrillar Aβ than for other the molecular imaging probes, such as PIB in FDG-PET [127]. Beyond labeling Aβ-plaques, Cur can also help to visualize the distinct morphology of different Aβ-plaques, such as core, neuritic, diffuse, and burned out plaques [13], indicating that it can be used to investigate the overall amyloid plaque loads, as well as an aid to characterize the morphology of Aβ-plaques after anti-amyloid therapy. Therefore, as a potent anti-amyloid polyphenol, Cur has a complete requisite profile for labeling and imaging the amyloid plaques.

### 6.2. Curcumin Therapy in Parkinson’s Disease

Parkinson’s disease (PD) is the second most common age-related neurodegenerative disease, and is characterized by bradykinesia, tremor, rigidity, and abnormalities in gait and posture. Gradual and selective degeneration of dopaminergic neurons in the substantia nigra pars compacta (SNpc), with a subsequent decline in dopamine (DA) levels in the nigro-striatal pathway are associated with PD [157,158]. Although most PD cases are sporadic, about 5% of the cases can be inherited. The major pathological hallmarks of PD are the presence of insoluble, fibrous aggregates, composed of α-syn in intraneuronal inclusions of Lewy bodies (LBs). In humans, PD is associated with the α-syn aggregation.

#### Effects of Cur on PD

**Figure 20:**
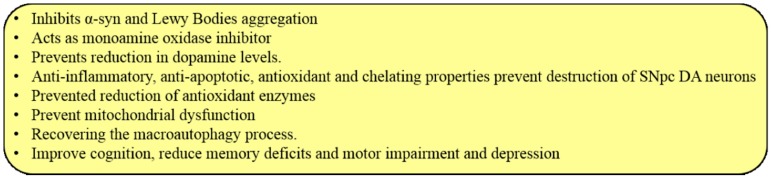
Pleotropic actions of Cur on Parkinson’s disease.

Cur has several beneficial effects on PD (Figure 20). First, Cur can inhibit α-syn aggregation and, prevent LB accumulation in vitro, attenuating α-syn oligomer toxicity in cells [35]. First, Cur reduces toxicity by binding to α-syn oligomers and fibrils (but not monomers), modulating the morphology, and reducing their aggregation, as shown by fluorescence and two-dimensional nuclear magnetic resonance (2D-NMR) studies [35]. Secondly, Cur attenuates reduction of DA levels, and degeneration of DA neurons [39]. Thirdly, Cur can reduce oxidative stress, memory deficits, and motor impairments [33]. Fourthly, Cur chelates iron, copper, and other metals, thus preventing α-syn or LB aggregation [159]. Fifthly, Cur promotes the recovery of macroautophagy by activating transcription factor EB, thus reducing cell death and neurotoxicity [160]. Sixthly, Cur can inhibit activity of monoamine oxidase (similar to MAO-inhibitor), thus restoring DA levels [161] and reducing depression [162]. Seventhly, Cur can protect DA neurons in brain by reducing ROS levels, maintaining mitochondrial functions, and attenuating neuroinflammation via CNB001, a Cur-derived compound [163] Finally, Cur can inhibit the JNK pathway and prevent dopaminergic neuronal loss via apoptosis [163] (Figure 20).

### 6.3. Curcumin in Huntington’s Disease

Huntington’s disease (HD), is a poly-glutamine (PolyQ) autosomal dominant genetic disorder characterize by progressive neurodegeneration and impairments of motor, psychiatric and cognitive functions [164]. Degeneration of medium spiny neurons of striatum, and cells in layers V and VI of cortex, SNpc, hippocampus, cerebellum, hypothalamus, and parts of the thalamus are the most commonly observed in HD [165,166]. The hallmark pathology in HD is the abnormal accumulation of misfolded mutated huntingtin protein (mHTT) as intracellular aggregates, which causes selective neuronal loss, primarily in the cortex and medium spiny neurons of striatum [165]. The Huntingtin gene (*HTT*) is present on the short arm of chromosome 4p16.3. HTT is ubiquitously expressed in neurons and is found in many subcellular compartments. Although its exact function is not fully understood, experimental animal studies have revealed that HTT is essential for fetal development and its absence is lethal [167]. HTT is involved in cytoskeleton anchoring, transport, cell signaling, and vesicle trafficking [168]. In addition, HTT upregulates the expression of brain derived neurotrophic factor (BDNF), inhibits caspase-3 and caspase-9, and protects against apoptosis [169]. A mutation in the HTT gene leads to expansion of the CAG (Cystocine-Adenine-Guanine) repeats which leads to elongation of polyQ in HTT protein and results in the accumulation of mHTT [170]. There is a correlation between the number of CAG repeats and the age of onset of the disease [165]. In general, more than 36 CAG repeats could trigger disease symptoms, and it is critical for its toxicity. Increase in the number of CAG repeats will cause greater HTT deposition, which increases neurotoxicity [170]. For example, animal models of HD, such as R6/1 and R6/2 mice or YAC128 mice, which express a larger CAG expansion, progressively develop cognitive, psychiatric and motor symptoms, which are analogous to those observed in HD patients [171,172,173,174]. Therefore, both loss of function in the wild type HTT and gain of function in the mutated form of HTT have been proposed to play a role in the development of HD pathology [169].

To date, the exact pathogenesis for the neuronal death in HD is not fully understood. However, glutamate excitotoxicity [175], mitochondrial dysfunction [176], impaired protein degradation, protein misfolding, caspase activation, transcriptional pathways dysregulation, decrease of proteasome function and abnormality in proteolysis are among the major causes for this disease [177].

#### Beneficial effects of Cur in Huntington Disease

**Figure 21:**
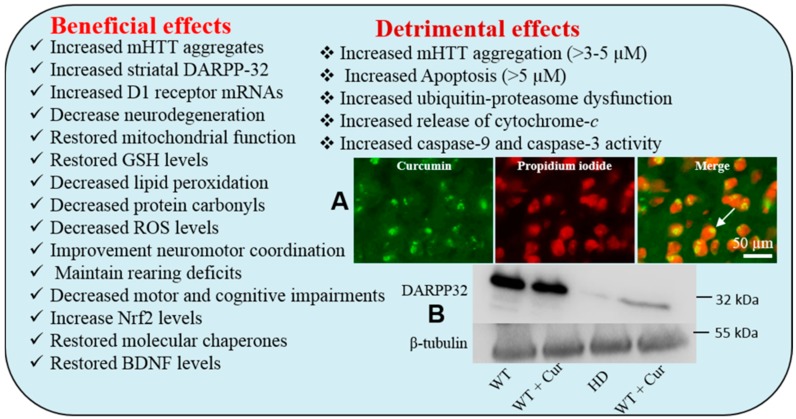
Effects of Cur on in vitro and in vivo models of HD. Several experiments have shown beneficial effects of Cur, but higher concentrations (>3–5 µM) of Cur may exacerbate HD symptoms. (**A**) The cortex of YAC128 mice was stained with Cur and images showed that Cur binds with HTT. (**B**) Western blot of DARP32, the marker for medium spiny neuron were partially restored by Cur treatment. Scale bar indicates 50 µm and is applicable to other images.

Cur has been shown to neuroprotective effects in animal models of HD. For example, when we fed chow containing 555 ppm Cur to CAG 140 knock-in HD mice, a significant decrease in mHTT aggregates and increased striatal DARPP-32 and D1 receptor mRNAs, as well as an amelioration of rearing deficits was observed [40]. Solid lipid nanoparticles (C-SLNs) given to treated 3-nitropropionic-acid (3-NP) treated rats (a toxin which causes HD-like neuropathology in rodents) resulted in a decrease of HD-like neurodegeneration, as well as significant increase in the activity of mitochondrial complexes and cytochrome levels [178]. Furthermore, C-SLNs also restored glutathione levels and superoxide dismutase (SOD) activity, decreasing lipid peroxidation, protein carbonyl formation, ROS levels, and mitochondrial swelling. In addition, C-SLN-treated rats showed significant improvements in neuromotor coordination, when compared with 3-NP-treated rats [178]. Furthermore, chronic treatment with Cur (10-, 20- and 50-mg/kg, once daily for 8 days, peritoneally) improved the motor and cognitive performances, reduced oxidative stress, and restored succinate dehydrogenase activity, which is inhibited in the in 3-nitropropionic acid (3-NP) HD rat model. An increase in Nrf2 (a key regulator for antioxidants enzyme expression) expression in C-SLN-treated mice, compared to controls, has also been observed [179]. Cur treatment also restored the down-regulated molecular chaperones (e.g. HSP40, HSP70) in HD. In addition, downregulated brain-derived neurotrophic factor (BDNF) in HD was also restored by Cur-treatments [180] (Figure 21).

In contrast, 3–5 µM of Cur can increase mHTT aggregation and mHTT-dependent cell death and promote proteasomal dysfunction in the mHTT expressing cells, in comparison to control cells [40,181,182]. Similarly, Jana and colleagues reported that Cur induced apoptosis through the impairment of the ubiquitin-proteasome system [183]. They have demonstrated that the exposure of Cur to the N2a cells causes a dose-dependent decrease in proteasome activity and an increase in ubiquitinated proteins. They concluded that the Cur can induce more apoptosis in proliferative cells than in differentiated cells, by decreasing the mitochondrial membrane potential, releasing cytochrome-*c* into cytosol, and activating caspase-9 and caspase-3 [183]. Although systemic availability of Cur is very low (~25 nM) after oral administration, it is assumed that low doses of Cur may not increase mHTT aggregation, but rather provides neuroprotective effects, but further study is needed to confirm this hypothesis.

### 6.4. Curcumin Therapy in Prion Diseases

Transmissible spongiform encephalopathies (TSE) or “prion” diseases are a group of rare, fatal, progressive neurodegenerative disorder which affect mammals [184]. It is a spectrum of diseases, which includes Creutzfeldt-Jakob disease, bovine spongiform encephalopathy, Scrapie, Kuru, Gersmann-Sträussler-Scheinker syndromes and fatal familial insomnia. In all these diseases the “prion” proteins are transmitted from cell to cell and induce accumulation of misfolded “prion” proteins (PrP) [184]. These accumulate as prion plaques, which can be observed throughout the CNS and cause neurodegeneration. Basically, prion aggregates generate several “holes” or “vacuoles” within the CNS, which make for a spongy neuronal architecture [185]. Prion disease can be genetic, sporadic, or infectious [186]. The PrP can take on an α-helix-rich, pathogenic, and cellular forms (PrP^C^), which can convert to a β-structure-rich, stable, insoluble, infectious, proteinase K-resistant fibril conformation of prion protein (PrP^Sc^) [184,185]. Although the mechanism of neuronal cell death in prion disease is unclear, the accumulation of this insoluble PrP^Sc^ is thought to be the principal reasons for dendritic or synaptic loss, as well as neurodegeneration, along with neuroinflammation in the CNS [184].

### 6.5. Effects of Cur on Prion Disease

Several drugs, small molecules or compounds have been tested for inhibiting PrP^Sc^ aggregation, but none of them have proven to be satisfactory for clinical use. Some recent research suggests that Cur can inhibit PrP^Sc^ fibril formation in scrapie-infected neuroblastoma (scNB) cells [38,41]. It can bind to non-native forms of PrP, thereby inhibiting prion fibril formation, without affecting native PrP [130]. Low doses of Cur (10 nM) can decrease ROS levels and effectively prevent PrP^Sc^-induced apoptosis [147].

## 7. Biphasic or Dose-Dependent Effects of Curcumin

Cur shows its biphasic or dose-dependent effects in our body, which means lower doses, show neuroprotection, whereas higher doses may be toxic to cells [147]. Several in vitro and in vivo studies suggest that Cur has beneficial effects, including potent antioxidant, anti-inflammatory, anti-amyloid properties at relatively lower doses (0.1–1 µM). Perhaps due to its limited bioavailability, Cur does not reach high concentrations in all tissues and organs. Therefore, it is assumed that the beneficial effects are due to low levels [147]. In contrast, higher doses (>5 µM) may induce colon or lung cancer or other side effects, such as inhibition of proteasomal function, which can occur at levels above a 3 µM concentration [183]. For example, ≥3 µM of Cur can increase mHTT-induced neurotoxicity in an in vitro model of HD [183]. It has been demonstrated that about 10 µg/mL of turmeric extract caused a dose- and time-dependent induction of chromosome aberrations, as well as DNA damage in several mammalian cell lines [187]. Even doses as small as 2.5–5 µg/mL of Cur have been shown to induce both mitochondrial- and the nuclear-DNA damage [188]. These reports raise a major concern about the safety of Cur therapy in different diseases. A recent report has also shown that Cur can promote lung cancer in mice [189].

**Figure 22:**
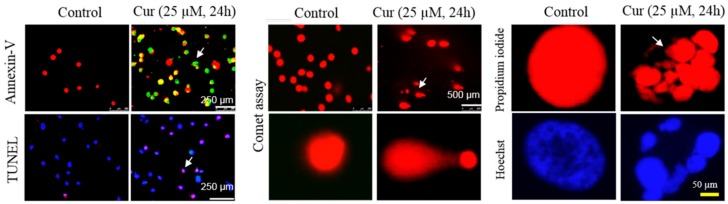
High concentrations of Cur can be used to kill glioblastoma cells. Cur treatment (25 µM) induces apoptosis and DNA fragmentation in human derived glioblastoma cell line (U-87Mg).

Interestingly, several studies also demonstrated that the higher concentration of Cur (5–50 µM) can be used to kill cancer cells, although it requires the cells to be treated with Cur for several hours or days [190]. For example, in one of our recent in vitro experiments, we treated glioblastoma cells (U-87 Mg, derived from human cells) with 25 µM of Cur for 24–72 h, and found a significant increase in DNA fragmentation and apoptotic death [14] (Figure 22). These data support many previous observations and suggest that higher concentrations of Cur can be used to kill cancer cells. Conversely, most therapeutic applications for Cur require low doses. However, when administered orally, achieving therapeutically significant concentrations of Cur outside the GI-tract is practically impossible because of its poor bioavailability. However, the Cur bioavailability in animal models of AD [12,13,108] and HD [40] following i.p. or oral administration was about 250–350 nM in the brain tissue. Also, when we imaged Cur-treated AD mouse brain tissue we observed that Cur was colocalized with Aβ profusely [13] and decreased Aβ plaques. This indicates that at least a few hundred-nM concentrations of unmodified Cur were available following either oral or i.p. administration of lipidated formula of Cur interact with Aβ, p-tau and prevent AD pathologies, such as neuroinflammation, thus reducing cognitive deficits [10,11,12,13]. Therefore, achieving optimal concentrations of Cur to the brain by oral administration while reducing the possibility of toxic effects to the periphery, needs to be carefully studied before widespread use of orally administered Cur can be advocated.

## 8. Recommended Doses and Limitations of Cur Therapy

To understand the beneficial roles of Cur, it is essential to evaluate its pharmacokinetics or pharmacodynamics after its administration in humans. Based on previous clinical studies, it has been demonstrated that 8 g per day of short-term Cur therapy has no significant detrimental effects [70]. Toxicological evaluations revealed that Cur is found to be pharmacologically safe, even up to 12 g per day, as reported by several animal studies and in phase-I clinical trials [32,70]. Similarly, another phase-1 human trial, with 8 g of Cur per day for three months, revealed no toxic effects [7]. However, a few studies have indicated that high doses of Cur can cause highly variable adverse side effects, including gastrointestinal discomfort, chest tightness, skin rashes, and swollen skin, as well as some allergic reactions, such as dermatitis [191] (Table 8).

In human studies with Cur, doses of 0.9–3.6 g per day for 1–4 months caused some adverse effects, including nausea and diarrhea, with an increase in serum alkaline phosphatase and lactate dehydrogenase [194]. Despite reports of possible adverse effects, most studies have revealed significant beneficial effects with Cur [12,30,39,45,46,57,58,69,135,144,150,160,182] (Table 9). For example, one of our previous studies with the 3XTg mouse model of AD showed that 600 nM is sufficient to reduce AD-like pathological symptoms [10,12]. Extrapolation of animal studies to clinical trials also revealed that an oral supplementation of Cur in the range of 80–500 mg per day was recommended to obtain these beneficial effects in humans, which means a daily intake of raw turmeric would be 2–4 g [10,126]. Furthermore, chronic intake of Cur sometimes may be hepatotoxic. Therefore, a person with liver diseases, such as cirrhosis, biliary tract obstruction, gallstones, obstructive jaundice and acute biliary colic, or those are under prescribed medication for hepatic problems are counter-indicated for Cur therapy, because Cur can stimulate bile secretion [74]. In fact, supplementation of even 20–40 mg of Cur per day can increase the gallbladder contractions in healthy people [195,196]. Similarly, alcoholics or heavy drinkers may not receive the benefits of this therapy. Furthermore, the individual taking any blood thinning agents, non-steroidal anti-inflammatory (NSAIDs) drugs, or reserpine are recommended not to take Cur, because, it can interact with these drugs [10,33,126]. However, for therapeutic purposes, dietary Cur is very unstable in most of the body fluids, and because of its poor water solubility and limited tissue bioavailability, it is highly recommended to mix Cur with oil or milk to enhance its absorption and metabolism [8]. Although the vast majority of animal studies and clinical trials using Cur has resulted in more beneficial than adverse effects [12,30,39,45,46,57,58,69,135,144,150,160,182], its use must be tempered by possible toxic effects at high doses.

## 9. Future Perspective of Curcumin Research

Numerous studies have been conducted to test the potential of Cur to prevent or treat different neurological diseases. However, several reports have raised questions about its safety and efficacy, especially at high doses, which may be harmful. Some researchers recommend limiting the daily intake of Cur to 1 g per day, whereas other studies have shown no side effects, even up to 12 g /day. However, our review of the studies using Cur for treating neurological disorders underscore a few critical observations. First, the formula of Cur can make a difference. Many researchers used whole turmeric extract, while others used different lipidated formulae of Cur, along with nano-Cur, and their data needs to be directly compared with the effects of natural Cur. In addition, the purity of Cur, along with a detailed description of the extraction method and sources are critical variables that need to be considered when making determination about the efficacy of Cur therapies. Second, the difference between Cur and curcuminoids needs to be considered. Many researchers use the term “curcuminoid” with Cur, but curcuminoid has two additional compounds (bis-methoxy and de-bismethoxy Cur). Therefore, this clarification needs to be considered when interpreting the result of such studies. Third, the mode of administration is important: It is critical to know how Cur is administered, because the PK and PD of Cur can vary with the route of administration, which can significantly affect the efficacy of the treatment. Fourth, the duration of Cur therapy can affect its efficacy: Both short- and long-term Cur therapy have shown mixed effects. Many scientists have reported short-term beneficial effects, whereas the potential of long-term studies to reveal toxic effects may be underreported, which underscores the need for more work using chronic administration of Cur. Overall, most scientists agree that Cur has enormous potential as an effective nutraceutical with advantages of having relatively low toxicity, being quite inexpensive, and easily obtained [12,30,39,45,46,57,58,69,135,144,150,160,182]. However, due to its poor bioavailability, different lipidated forms continue to be developed, which is providing increasingly greater bioavailability and efficacy.

## 10. Conclusions

Neurodegenerative diseases are age-related complicated disorders with complex neuropathological characteristics. They develop progressively, and oftentimes significant neuropathology precedes any overt clinical symptoms. Neuronal damage and cognitive deficits or impairment of motor coordination are the major problems in these diseases. Because of its pleotropic actions on the nervous system, including anti-amyloid, anti-inflammatory, and anti-oxidant properties, Cur is a promising candidate for targeting protein misfolding neurological diseases. Furthermore, it is safe and inexpensive, readily available and can effectively penetrate the blood-brain barrier and neuronal membranes. We have provided detailed information on the anti-amyloid properties of Cur in major neurodegenerative disorders, such as AD, PD, HD, and prion diseases. Collectively, the information available from reviewing the literature on the therapeutic potential of Cur can provide helpful insights into the potential clinical utility of Cur for treating neurological diseases.

## Figures and Tables

**Table 1:** The chemical and biophysical properties of curcuminoid [15].

Characteristics	Cur-I	Cur-II	Cur-III
Common Name	Cur	DemethoxyCur	BisdemethoxyCur
Chemical Name	Dicinnamoyl methane	4-OH cinnamoyl methane	Bis-4-OH cinnamoyl methane
Color	Bright orange-yellow	Bright orange-yellow	Bright orange-yellow
Amount Present (%)	77	17	3
Molecular Mass (g/mol)	368.4	338.0	308.1
Melting Point (°C)	183.0–186.0	172.5–174.5	224.0
Neutral Solvent (water)	Poorly soluble	Poorly soluble	Poorly soluble
Solubility in Organic Solvents	Soluble	Soluble	Soluble
Solubility in Hexane or Ether	Insoluble	Insoluble	Insoluble
Excitation/Emission in	420/530 nm	420/530 nm	420/530 nm
Excitation/Emission in Alcohol	536–560 nm	Unknown	Unknown

**Table 2:** Anti-amyloid activities of Cur in major neurodegenerative diseases.

Proteins	Diseases	Nature of Binding of Cur	Outcomes	Ref.
Aβ	AD	With amino acid 16–21 of Aβ	Inhibits oligomer and fibril formation, thus decrease Aβ induced neurotoxicity	[9,13,34]
Tau	Tauopathies, AD	In the microtubule-binding region of tau	Inhibits phosphorylated tau, thus decrease neurofibrillary tangle	[12,37]
α-Syn	PD	In the hydrophobic no Aβ component region	Inhibits α-syn oligomers and fibril formation, thus decrease α-Syn induced oxidative damage	[35,39]
HTT	HD	Unknown	Lower doses (nM) decrease HTT aggregates	[36,40]
Prion	Prion	α-Helical intermediate and to the amyloid form of prion protein	Inhibits PrP^sc^ accumulation	[38,41]

**Table 3:** Different components used to increase Cur solubility and bioavailability. Scientists used different materials, including adjuvants, proteins, lipid nanoparticles, and synthetic materials to increase Cur solubility [75].

Materials	Compounds Used with Cur
Adjuvant	Piperine
Bio-conjugates	Turmeric oil, glycine, alanine, EGCG
Lipids	Phospholipid, liposome, oil body emulsion
Nanoparticles	GMO, Chitosan, cyclodextrin, PLGA, silica, PHEMA, gold, silver, casein, orange gel-based nano emulsion, dendrimer, solid lipid particles
Protein	BSA, soy protein isolated
Others	Hyaluronic acid, hydrogel, polymer, PEG-PEI emulsion, polymer encapsulated, beta-lactoglobulin

**Table 4:** Average values of pharmacokinetic parameters of Cur-lecithin-piperine and BCM-95^®^ (Biocurcumax™).

Parameters	Tmax	Cmax	Ke	t1/2	AUC (o.inf)	Cl (Observed)/F	Vz (observed)/F
**Cur**	2	149.8	0.296	2.63	461.86	0.006735	0.026362
**BCM—95 RCG**	3.44	456.88	0.26	4.96	3201.28	0.001682	0.006784

Tmax: Time of peak plasma concentration, Cmax: Peak plasma concentration, Ke: Elimination rate constant, t1/2: Half-life, AUC (0-infinity): Area under curve from ‘0’ h to infinity, Cl/F: Clearance/Bioavailability, Vd/F: Volume of distribution/bioavailability [77].

**Table 5:** Nanoparticles-conjugated Cur formulae, characteristics and their biological effects [11].

Nanoparticles	Schematic Diagram	Shape	Size (nm)	Methods	Outcome
Liposome	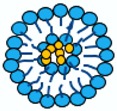	Globular	25–205	In vitro, in vivo (dogs & mice)	Increased solubility, tissue distribution, and stability
Micelles	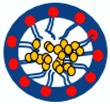	Spherical	10–100	In vitro, In vivo (mice)	Increased solubility and bioavailability; Improved anti-oxidative properties
Noisome	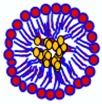	Lamellar	190–1140	In vitro, In vivo (snake and mice)	Increased skin penetration; Prolonged delivery system
Nanogel network	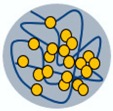	Cross-linked polymer	10–200	In vitro	Increased stability, fluorescence effects, developed bioavailability, get better control release; Prolonged half-life
Chitosan	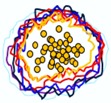	Linear polysaccharide composed	100–250	In vitro, In vivo (rats and mice)	Improved chemical stability, improved antioxidant effects; Prolonged blood circulation
Gold	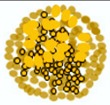	Globular	200–250	In vitro	Improved solubility; Enhanced antioxidant
Silver	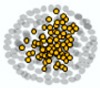	Film layer	~15	In vitro	Improved wound healing; Increased antiviral and anticancer effects
Cyclodextrin	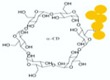	cyclic oligomers of glucose oligosaccharide		In vitro	Improve stability and bioavailability of Cur
Dendrimer	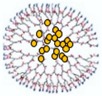	Globular polymer	15–150	In vitro, In vivo (mice)	Improved stability; Increased antitumor and anti-proliferative effects
Solid lipid	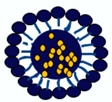	Spherical	50–1000	In vitro, In vivo (rat and mice)	Prolonged circulation of blood; Increased anti-inflammatory effects; Improved brain delivery

**Table 6:** Pleotropic actions of Cur for AD therapy. Among them, anti-amyloid, anti-oxidant and anti-inflammatory activities are considered the most promising for treating AD [33,126].

Actions	Mechanisms	References
Anti-amyloid properties	Binds with Aβ and prevent its oligomerization and fibril formation	[9,112,127]
Inhibition of Aβ production	Inhibits activities of β-secretase (BACE), inhibiting amyloid precursor protein (APP) processing pathway	[33,128]
Aβ clearance	Stimulates phagocytosis, thus decrease Aβ-plaques	[9,10,51]
Inhibition of NFTs	Binds with NFTs and inhibits tau phosphorylation (pTau)	[129]
Inhibition of other amyloids	Binds with α-synuclein in PD, huntingtin in HD, and prion aggregates in prion diseases	[35,130]
Potent antioxidant	Scavenges ROS/RONS, increase antioxidant levels, decreases lipid peroxidation, chelates toxic metals	[10,51,131]
Anti-inflammatory activities	Downregulates NF-κB, COX-2, 5-LOX, TNFα, IL-1, IL-6.	[10,51]
Regulates activity of molecular chaperones	Restores levels of heat shock proteins (HSP90, 70, 60, 40, HSC70), proteasome system	[132]
Enhance NGF, BDNF, GDNF, neurogenesis and synaptogenesis	Increase expression of BDNF, NGF, GDNF and can promote neurogenesis, and synaptogenesis	[10,133]
Improving cerebral circulation	Inhibits inflammation of brain vasculature leading to improvement of overall blood supply, reduces platelet adhesion in the brain microvascular endothelial cells	[69,134]

**Table 7:** Curcumin therapy in different animal models and their outcomes [135].

Animal Models	Dose and Duration of Treatment	Disease	Outcomes	Ref.
Sprague-Dawley rat	Diet, 500 and 2000 ppm, 2 months	AD (Aβ ICV infusion)	Decrease spatial memory deficit, oxidative damage, microgliosis	[135]
3XTg-AD mice	Diet, 555 ppm, 2 months	AD (Aβ overexpression)	Decreased Aβ plaque deposition	[12]
APPswe/PS1dE9 mice	Diet, 160 and 5000 ppm, 6 months	AD (Aβ overexpression)	Reduced hippocampal Aβ40/Aβ42 levels	[136]
APPswe/PS1dE9 mice		AD (Aβ overexpression)	Improved spatial memory and decreased Aβ40/Aβ42 levels	[114]
Tg2576 mice	Diet, 500 ppm, 4 m	AD (Aβ overexpression)	Decrease cell death, Aβ-plaques, prevent fibril formation	[9]
PS-1dE9 mice	IV, 7.5 mg/kg/day, 7 days	AD (Aβ overexpression)	Increased restoration of distorted neuritis, plaque disruption	[135]
Kunming mice	PO, 200 mg/kg, 45 days	AD (AlCl3, d-galactose)	Decrease spatial memory deficit	[135]
Sprague-Dawley rat	PO, 50 mg/kg, 4 days	PD (6-OHDA)	Improve TH+ cell numbers	[135]
ICR mice	IP, 50 mg/kg, 3 times	PD (MPTP)	Decreased oxidative damage, increase dopaminergic neurons	[137]
Swiss albino mice	IP, 80 mg/kg, 7 days	PD (MPTP)	Decreased MAO-B	[135,138]
CAG140 mice	Diet, 555 ppm, 2m	HD (knock in)	Decreased huntingtin aggregation, increase rearing, decrease climbing	[40]
5XFAD	IP, 100 mg/kg, 2–5 days	AD (transgenic)	Decreased Aβ plaque, prevent cell death	[108]

**Table 8:** Side effects of Cur therapy when intake orally.

Parameter	Side Effects	Ref.
General effects	Gastrointestinal discomfort, chest tightness, skin rashes, and swollen skin, allergic reactions or dermatitis, nausea, and diarrhea	[192]
Blood clotting	Slow down blood clotting process	[193]
Gall bladder	Increase gallstones contraction and increase bile duct obstruction	[192]
Pregnancy and postnatal complications	Stimulate the uterus or promote a menstrual period. Breast feeding women not recommended	[8]
Stomach problems	Increased stomach acid secretion if taken with antacid drugs	[8]

**Table 9:** List of a few clinical trials with Cur in Alzheimer’s disease.

Study ID	Curcumin Molecule	Cohort	Dose	Duration	Outcomes	Ref.
Baum et al. NCT00164749	Cur + gingko	AD:50+ year, *n*= 30	1, 4 g/day	6 months	No differences in Aβ levels between treatments or MMSE scores	[71]
Ringman et al. (ACT00099710)	Cur C3 complex	Mid/Moderate AD, 49 y+, *n* = 30	2, 4 g/day	24 weeks	No differences detected between treatment groups in biomarkers measured, low bioavailability	[197]
Hishikawa et al.	Tumeric capsule	Severe AD, *n* = 3	100 mg/day	12 months tested after 12 weeks	MMSE and NPIQ; score on NPIQ decreased significantly, MMSE increased in 1/3	[198]
Poncha (NCT01001637)	Longvida	Moderate-severe AD, 50–80 y, *n* = 160	2, 3 g/twice daily	2 months	Efficacy and safety: blood and cognition	[199]
Martin and Goozee (ACTRN12613000681752)	Biocurucmax (BCM-95)	Retirement, healthy, 65–95, *n* = 100	500 mg/thrice/day	12 months	Cognition, blood biomarkers, brain imaging, retinal imaging	[199]
Martin (ACTRN12611000437965)	Biocurucmax (BCM-95)	Community living, healthy, 55–75, *n* = 100	500 mg/thrice/day	12 months	Cognition, blood biomarkers, life style, brain imaging	[199]
Small et al. (NCT01383161)	Tetracurcumin CR-031P^TM^	MCI, normal aging, *n* = 132	90 mg/twice/day	18 months	Cognition, blood genetic profile	[200]
Frautschy (NCT018811381)	Longvida and Yoga	Subjective cognitive complainers, 55–90, *n* = 80	400 mg/twice/daily	6 months	Biochemistry, cognition, brain imaging	[199]
Cox et al. (ACTRN12612001027808)	Longvida^TM^	Healthy and cognitive decline, 65–80, *n*= 60	400, 800 mg/daily	4weeks 8 weeks	Cognition, mood and anxiety, blood biomarkers, MRI	[201]
NCT00595582	Curcumin bioperine	MCI, 55–85, *n* = 10	900 mg/twice/daily	24 months	Cognition and size of metabolic lesion by PET	[199]
ACTRN12614001024639	BCM-95	Healthy and MCI, 65–90 years, *n* = 48	500 mg/twice/daily	3 months	Gene regulation and expression, and cognition	[199]
ACTRN12613000367741	Longvida^TM^	Healthy, MCI, mild/moderate AD, 50 years, *n* = 200	20 g/daily	7 days	Diagnostics, Curcumin fluorescent retinal imaging of Aβ plaques	[199]

## Abbreviations

Cur	Curcumin
CNS	Central nervous system
AD	Alzheimer disease
SLN	Solid lipid nanoparticles
DMC	Demethoxycurucmin
BDMC	Bisdemethoxycurucmin
IUPAC	International Union of Pure and Applied Chemistry
DMSO	Dimethyl-sulfoxide
DMF	Dimethyl formamide
PK	Pharmacokinetics
PD	Pharmacodynamics
THC	Tetrahydrocurcumin
HHC	Hexahydrocurcumin
i.v.	Intravenously
i.p.	Intraperitonally
OHC	Octahydrocurcumin
Aβ	Amyloid β-protein
α-syn	Alfa-synuclein
HTT	Huntingtin
PD	Parkinson’s disease
HD	Huntington’s disease
p-tau	Phosphorylated tau
BBB	Blood brain barrier
PUFA	Polyunsaturated fatty acids
ROS	Reactive oxygen species
GSH	Glutathione (reduced)
SOD	Superoxide dismutase
GPx	Glutathione peroxidase
GST	Glutathione S-transferase
NF-κB	Nuclear factor κ-light-chain-enhancer of activated B cells transcription factor
COX	Cyclooxygenase
LOX	Lipoxygenase
TNF	Tumor necrosis factor
IL	Interleukin
PPARγ	Peroxisome proliferator-activated receptor gamma
iNOS	Induced nitric oxide synthase
HSP	Heat shock protein
NGF	Nerve growth factor
BDNF	Brain derived neurotropic factor
GDNF	Glial derived neurotropic factor
PDGF	Platelet derived neurotropic factor
PSD	Post-synaptic density protein
HAT	Histone acetyltransferase
BMECs	Brain microvascular endothelial cells
GI	Gastrointestinal
EGCG	Epigallocatechin gallate
GMO	Genetically modified organism
PLGA	Poly lactic-*co*-glycolic acid
BCM-95	Biocurcumax-95
IC	Inhibitory concentration
DLS	Dynamic light scattering
SEM	Scanning electron microscope
FTIR	Fourier transforms infrared spectroscopy
ER	Endoplasmic reticulum
AuNPs	Gold nanoparticles
AgNPs	Silver nanocomposite
Cds	Cyclodextrins
SLNP	Solid lipid nanoparticles
nm	Nanometer
nM	Nanomolar
ppm	Parts-per-million
SLCP	Solid lipid Cur particle
NFT	Neurofibrillary tangle
BACE	β-secretase
APP	Amyloid precursor protein
PHF	Pair helical filaments
GSK-3β	Glycogen synthase kinase-3β
MAPK	Mitogen-activated protein kinase
Cdk5	Cyclin-dependent kinase 5
ERK2	Extracellular signal-regulated kinase 2
MARK	Microtubule affinity-regulating kinase
SADK	SAD-kinase
PKA	Protein kinase A
CaMKII	Calcium/calmodulin-dependent protein kinase II
JNKc	Jun N-terminal kinase
IRS	Insulin receptor substrate
GFAP	Glial fibrillary acidic protein
Iba-1	Ionized calcium-binding adapter molecule 1
PET	Positron emission tomography
NIR	Near infrared
PIB	Pittsburgh compound B
FDG	2-Deoxy-2-[18F] fluoroglucose
SNpc	Substantia nigra pars compacta
DA	Dopamine
LBs	Lewy bodies
2D-NMR	Two-dimensional nuclear magnetic resonance
MAO	Monoamine oxidase
PolyQ	Poly-glutamine
mHTT	Mutated huntingtin protein
CAG	Cytosine-adenine-guanine
YAC	Yeast artificial chromosome
DARPP	Dopamine-and cAMP-regulated neuronal phosphoprotein
TSE	Transmissible spongiform encephalopathies
PrP	Prion protein
PrP^C^	Cellular form of prion protein
NSAIDs	Non-steroidal anti-inflammatory drugs
NPI-Q	Neuropsychiatric Inventory–Questionnaire
PBS	Phosphate Buffer Saline
